# High Potential for Secondary Metabolite Production of *Paracoccus marcusii* CP157, Isolated From the Crustacean *Cancer pagurus*

**DOI:** 10.3389/fmicb.2021.688754

**Published:** 2021-06-28

**Authors:** Janina Leinberger, Jonas Holste, Boyke Bunk, Heike M. Freese, Cathrin Spröer, Leon Dlugosch, Anna-Carlotta Kück, Stefan Schulz, Thorsten Brinkhoff

**Affiliations:** ^1^Institute for Chemistry and Biology of the Marine Environment, University of Oldenburg, Oldenburg, Germany; ^2^Institute of Organic Chemistry, Technische Universität Braunschweig, Braunschweig, Germany; ^3^Leibniz-Institute DSMZ, German Collection of Microorganisms and Cell Cultures, Braunschweig, Germany

**Keywords:** *Paracoccus*, *Rhodobacteraceae*, growth inhibition, genome analysis, natural products, bioactivity, antibiosis, microbial interactions

## Abstract

Secondary metabolites are key components in microbial ecology by mediating interactions between bacteria and their environment, neighboring species or host organisms. Bioactivities can be beneficial for both interaction partners or provide a competitive advantage only for the producer. Colonizers of confined habitats such as biofilms are known as prolific producers of a great number of bioactive secondary metabolites and are a potential source for novel compounds. We investigated the strain *Paracoccus marcusii* CP157, which originates from the biofilm on the carapace of a shell disease-affected *Cancer pagurus* specimen, for its potential to produce bioactive secondary metabolites. Its closed genome contains 22 extrachromosomal elements and several gene clusters potentially involved in biosynthesis of bioactive polyketides, bacteriocins, and non-ribosomal peptides. Culture extracts of CP157 showed antagonistic activities against bacteria from different phyla, but also against microalgae and crustacean larvae. Different HPLC-fractions of CP157 culture extracts had antibacterial properties, indicating that several bioactive compounds are produced by CP157. The bioactive extract contains several small, antibacterial compounds that partially withstand elevated temperatures, extreme pH values and exposure to proteolytic enzymes, providing high stability toward environmental conditions in the natural habitat of CP157. Further, screening of 17 *Paracoccus* spp. revealed that antimicrobial activity, hemolysis and production of *N*-acyl homoserine lactones are common features within the genus. Taking into account the large habitat diversity and phylogenetic distance of the tested strains, we hypothesize that bioactive secondary metabolites play a central role in the ecology of *Paracoccus* spp. in their natural environments.

## Introduction

Among other processes, secondary metabolites mediate antagonistic or mutualistic interactions and are key components in microbial ecology (Seyedsayamdost et al., 2011; Beyersmann et al., 2017). Increased production of bioactive metabolites often correlates with strong competition within habitats restricted in availability of nutrients or space (Molloy and Hertweck, 2017; Mullis et al., 2019). Especially marine epibiotic bacteria were identified as prolific producers of a broad range of antagonistic compounds. Some substances are excreted to gain direct competitive advantage over neighboring species, or mediate interactions with a host organism by protection from fouling organisms or settlement of pathogens (Egan et al., 2013; Molloy and Hertweck, 2017). However, host-bacteria interactions are not always mutually beneficial. Seyedsayamdost et al. (2011) demonstrated that *p*-coumaric acid, which leaches from aging microalgae, triggers production of algicidal compounds in *Phaeobacter inhibens*. Consequently, a chemically induced shift of *P. inhibens*’ lifestyle to opportunistic pathogenicity leads to rapid release of large amounts of nutrients via algal lysis and eventually detachment from the dying host.

Epibiotic microbial communities on the carapace of the arthropod *Cancer pagurus* (CP) were analyzed in several previous studies, reporting presence of bacteria mainly belonging to the phyla *Actinobacteria*, *Bacteroidetes*, and *Proteobacteria* (Quinn et al., 2012; Kraemer et al., 2020). British, Irish, and German populations of *C. pagurus* were found to be heavily affected by the “black spot” shell disease (Wootton et al., 2014; Kraemer et al., 2020). Characteristic symptoms are progressive melanization and degradation of the exoskeleton (Chistoserdov et al., 2012). Most authors assume a polyphasic development that involves initial damage of the non-chitinous outermost surface layer (epicuticle) and subsequent degradation of the chitinous inner layer (procuticle) (Vogan et al., 2002). Both processes are linked to changes in the arthropods microbiome, bacterial degradation via chitinolytic bacteria and secondary colonization by opportunistic pathogens (Smolowitz et al., 2005). In a recent study, 75 new strains were isolated from the biofilm on the carapaces of “black spot” shell disease-affected *C. pagurus* specimens (Bergen, 2018). Several bacterial isolates are affiliated with the genus *Paracoccus*, among which strain *Paracoccus marcusii* CP157 was obtained from a black spot. CP157 corresponds to an operational taxonomic unit (OTU) that made up >1% of the bacterial community in the respective sample, while the same OTU was present, but less abundant, in unaffected areas of the carapace. Thus, CP157 is assumed to play a role in the development or progression of black spot shell disease in *C. pagurus*. Even though *Paracoccus* is one of the largest genera within the *Rhodobacteraceae*, and despite great interest in secondary metabolism of other members of this family, details on biosynthesis and modes of action of bioactive secondary metabolites produced by *Paracoccus* are scarce.

So far, the genus *Paracoccus* was studied mainly because of its versatile primary metabolism (Li et al., 2020). *Paracoccus* spp. have been isolated from diverse marine and terrestrial habitats including associations with insects, corals and bryozoans (Lasek et al., 2018). This ability to thrive in different environments is most likely based on high metabolic flexibility, a general feature of the *Rhodobacteraceae* (Brinkhoff et al., 2008), including production of bioactive secondary metabolites. Experimental and genomic approaches revealed that *Paracoccus* sp. Arc7-R13 is capable of extracellular synthesis of silver nanoparticles, when incubated with silver nitrate (Zhang et al., 2019; Li et al., 2020). These particles have strong antibacterial activity against strains of *Escherichia coli*, *Pseudomonas aeruginosa*, *Bacillus subtilis*, and *Staphylococcus aureus*. Other studies reported antibacterial activity of *Paracoccus* sp. S4493 and of culture extracts prepared from *Paracoccus pantotrophus* FMR19 (Machado et al., 2015; Faridha Begum et al., 2016). Zhang et al. (2018) found algicidal activity against the dinoflagellate *Prorocentrum donghaiense* in *Paracoccus* sp. Y42, and Lee et al. (2012) reported activity of *Paracoccus* sp. PC101 against *Candida albicans*. Moreover, several studies described complex structures of *Paracoccus* genomes with multiple extrachromosomal elements and putative gene clusters involved in the synthesis of bioactive secondary metabolites (Dziewit et al., 2012; Maj et al., 2013; Lasek et al., 2018). However, no comprehensive investigation of the potential of *Paracoccus* spp. to produce bioactive secondary metabolites has been conducted. In this study, we reveal the potential for secondary metabolite production of the new strain *P. marcusii* CP157, based on genomic and physiological characteristics. To identify whether the detected features are specific adaptations of CP157 or common traits within the genus, we compared the results for CP157 to further 17 *Paracoccus* spp. that originate from other habitats such as soils, sea water, or associations with higher organisms.

## Materials and Methods

### Origin and Cultivation of Organisms

#### Source of Strains

*Paracoccus marcusii* CP157, used here as a model strain to study its potential to produce bioactive secondary metabolites, originates from a diseased spot on the carapace of a CP specimen. Further 17 *Paracoccus* spp. from diverse habitats (Supplementary Table 1) were screened for production of secondary metabolites. Nine of these *Paracoccus* spp. were obtained from culture collections and are type strains of the respective species. Bioactivity of bacteria and their culture extracts were tested against bacterial target strains from four different classes, i.e,. Actinobacteria, Alphaproteobacteria, Bacilli, Flavobacteriia as well as the two diatoms *Skeletonema marinoi* CCMP 1332, *Thalassiosira rotula* CCMP 1647 and larvae of the crustacean *Artemia salina*. An overview of all organisms used in this study and their origin is given in Supplementary Table 1.

#### Routine Cultivation of Organisms

All bacterial strains were routinely cultivated in marine broth (MB) medium with modifications given by Beyersmann et al. (2017). Only the type strain of *Paracoccus alcaliphilus* was grown in DSMZ medium 772^1^. If not stated otherwise, incubations were carried out in in the dark at 20°C and 100 rpm. Growth was monitored by measuring the optical density at 600 nm (OD_600 *nm*_). Agar plates were prepared with 1.7% (w/v) agar and incubated at room temperature. For cultivation of bacteria in defined medium, artificial sea water medium (ASW, Zech et al., 2009) was supplemented with a single carbon source. Substrates were sterile filtered and added after autoclaving. Adaptation of bacteria to defined medium was achieved via two transfers: the first pre-culture was set up in ASW + MB (10%). Subsequently, cells were transferred to ASW + substrate and incubated for 2 days before inoculation of the main cultures.

Axenic cultures of diatoms were cultivated without shaking at 15°C in a 12 h light/12 h dark diurnal cycle in another artificial sea water medium (ESAW, Harrison et al., 1980). Algal growth was monitored by measuring autofluorescence of chlorophyll in a microplate reader (Spark, Tecan, Männedorf, Switzerland) at 488 nm excitation and 680 nm emission wavelength.

*Artemia salina* larvae were hatched from a ready-to-use mixture of eggs, sea salts, and microalgae (ArtemioMix, JBL GmbH & Co. KG, Neuhofen, Germany). Subsequently, larvae were transferred to a 12-well plate containing 3 ml ESAW and algal culture for nutrient supply.

#### Sequencing and Analysis of the *Paracoccus marcusii* CP157 Genome

##### Genome sequencing of CP157

DNA was isolated from CP157 using a Qiagen Genomic-tip 100/G (Qiagen, Hilden, Germany). A SMRTbell^TM^ (PacificBiosciences, Menlo Park, CA, United States) template library was prepared according to the manufacturer’s instructions. Briefly, for preparation of 15 kb libraries 5 μg genomic DNA were end-repaired and ligated overnight to hairpin adapters applying components from the DNA/Polymerase Binding Kit P6 (Pacific Biosciences, Menlo Park, CA, United States). Reactions were carried out and BluePippin^TM^ Size-Selection was performed as recommended by the manufacturer (Sage Science, Beverly, MA, United States). Conditions for annealing of sequencing primers and binding of polymerase to purified SMRTbell^TM^ template were assessed with the Calculator in RS Remote. SMRT sequencing was carried out on the PacBio *RSII*, taking one 240-min movie on a single SMRT cell, resulting in 58,447 post-filtered reads with a mean read length of 5,392 bp. Libraries for sequencing on the Illumina platform were prepared with the Nextera XT DNA Library Preparation Kit, with modifications (Baym et al., 2015), and sequenced on the Illumina MiSeq^TM^ (Illumina, San Diego, CA, United States), leading to 566,300 Mio reads of 2 × 300 bp. Long read genome assembly was performed with the “RS_HGAP_Assembly.3” protocol included in SMRTPortal version 2.3.0 using default parameters, with exception of the target genome size, which was increased to 10 Mbp. In order to screen for additional plasmids, an independent short read assembly was performed using Trimmomatic (Bolger et al., 2014) and SpaDES 3.14 (Bankevich et al., 2012). The replicons were circularized, particularly artificial redundancies at the ends were removed and adjusted to the replication genes. Identification of redundancies and replication genes was done via BLAST. Circularization and rotation to the replication genes was performed by genomecirculator.jar tool^2^. Error-correction was performed by mapping Illumina short reads onto finished genomes using Burrows-Wheeler Alignment bwa 0.6.2 in paired-end mode using default settings (Li and Durbin, 2009), with subsequent variant and consensus calling using VarScan 2.3.6 (Koboldt et al., 2012). Genome annotation is based on Prokka 1.13 (Seemann, 2014) with subsequent manual curation. The complete genome is deposited in GenBank under the accession numbers CP065892-CP065914. The genome of *P. marcusii* DSM 11547T was sequenced on the Illumina MiSeq^TM^ as described above for means of taxonomic delineation only. Raw reads were deposited in NCBI SRA under accession number PRJNA693451.

##### Analysis of the CP157 genome and identification of secondary metabolite biosynthesis gene clusters

Assignment of COG categories was achieved using the eggNOG-mapper v2 (Huerta-Cepas et al., 2019). For detection of biosynthetic gene clusters (BSGCs) the online tool antiSMASH 5.2 (Blin et al., 2019) was applied. Settings were kept in default and the algorithm was run in strict mode. The obtained hits were compared with existent entries for *Paracoccus* spp. in the antiSMASH database. Genes identified as core biosynthetic genes were further investigated by pBLAST of the amino acid sequence. To identify potential regions of horizontal origin or putative genomic islands, the genome of CP157 was uploaded to IslandViewer 4 (Bertelli et al., 2017). Regions containing prophages or remnants of such were identified with PHASTER (Arndt et al., 2016). Screening for gene transfer agents (GTAs) was done via BLAST against known GTA genes. Therefore, the respective amino acid sequences of GTA genes from *Paracoccus seriniphilus* (Ga0192381_102327; Ga0192381_12519), *Paracoccus* sp. S4493 (Ga0308526_100156; Ga0308526_103914), *Paracoccus* sp. PAMC 22219 (Ga0098299_100157), and *Dinoroseobacter shibae* DFL-12 (Dshi_2174) were downloaded from IMG/M (Integrated Microbial Genomes and Microbiomes) and used as query sequences for pBLAST against the CP157 genome.

## Phylogenetic Analysis and Taxonomic Identification

*Paracoccus* strains used in this study were identified based on their 16S rRNA gene sequence similarity. Sequences were obtained from the NCBI nucleotide database^3^. Phylogenetic analysis of 16S rRNA gene sequences was performed using the ARB software package (Ludwig et al., 2004). Due to high sequence similarity between the 16S rRNA genes of CP157 and related strains, *in silico* DNA–DNA hybridization (DDH) was performed using the Genome-to-Genome Distance Calculator (CGDC) 2.1 with formula 2^4^. For taxonomic delineation, reference genomes of the best hits obtained from nBLAST of the 16S rRNA gene of CP157 were downloaded from GenBank. A draft genome was available for the type strain *Paracoccus hibiscisoli* CCTCC AB2016182 (GCA_005048265.1). Due to their high 16S rRNA gene sequence similarity, the strains *Paracoccus haeundaensis* CGMCC 1.8012 (GCA_006239215.1) and *Paracoccus* sp. Arc7-R13 (GCA_004010775.1) were included in the analysis. For optimal taxonomic resolution, the genome of the closest described relative *P. marcusii* DSM 11574T was newly sequenced and included in DDH. GGDC was run with default settings.

### Physiological Characterization of CP157 and Screening for Bioactive Metabolites in Different *Paracoccus* spp.

#### Bioassays for Detection of *N*-acyl-homoserine lactones and Hemolysins

The biosensor strain *Agrobacterium tumefaciens* NTL4 (pZLR4) was used to detect the release of AHLs by the tested *Paracoccus* spp. The bioassay was carried out as described (Ravn et al., 2001), with minor modifications. In brief, an overnight culture of *A. tumefaciens* was inoculated in LB medium and transferred to ABT medium (both supplemented with 25 μg/ml gentamicin). ABT agar [1.2% (w/v)] was prepared according to Ravn et al. (2001), with 50 μg/ml X-Gal (5-bromo-4-chloro-3-indolyl-β-D-galactopyranoside) and 25 μg/ml gentamicin, and seeded with *A. tumefaciens* pre-culture directly before pouring the plates. Wells were punched out of the agar and filled with 50 μl *Paracoccus* spp. pre-culture (MB; exponential phase). After incubation at room temperature for 24–48 h, color development was noted as indication for AHL production.

To screen for hemolytic activity, Columbia agar plates with 5% sheep blood (Fisher Scientific GmbH, Schwerte, Germany) were streak-inoculated with 50 μl *Paracoccus* spp. pre-culture (MB). Plates were incubated at room temperature for 14 days and examined daily for degradation of red blood cells.

#### Antibacterial, Antilarval, and Antialgal Activity

##### Preparation of crude extracts

*Paracoccus* spp. were grown in 300 ml MB supplemented with 2 g/100 ml of the adsorbent Amberlite XAD (Sigma-Aldrich, St. Louis, MO, United States). Two different types, XAD-7 (moderately polar) and XAD-16 (non-polar), were applied for each strain and bioactivities of the resulting extracts were compared. If not stated otherwise, the adsorbent was always recovered from the cultures in the late stationary growth phase by filtration and eluted with methanol (MeOH). The methanolic extract was dried in a rotary evaporator (Multivapor *P*-6, Büchi, Flawil, Switzerland) and stored as a powder at −20°C until further use.

##### Antibacterial activity of Paracoccus spp.

Crude extracts were tested for antibacterial activity in a modified disk diffusion assay developed by Bauer et al. (1966). All culture extracts were tested for activity against *Paracoccus* spp. CP32 and CP35, *Arthrobacter* sp. CP30 and *Aquimarina* sp. CP51. The additional target strains mentioned in Supplementary Table 1 were exposed to culture extracts of CP157 to determine the range of activity. The test was performed by homogenously spreading 200 μl culture of the target strain (adjusted to OD_600 *nm*_ = 0.03) on agar plates. Filter paper flakes were soaked with 50 μl of culture extract (50 mg/ml). Negative controls of methanol and extracted MB had no inhibitory effect, which ruled out any negative impact of solvent or culture medium components on the growth of the target strains. The paper flakes were placed on the inoculated test plate and checked for development of inhibition zones in the following 24–48 h. The test was considered positive when colony-free halos were visible with the unaided eye around the paper flakes. Results are either expressed in (mm) radius around paper flakes or as arbitrary units (AU) by dividing the mean size of inhibition zone by the extracted volume and multiplying with 100.

##### Impact of CP157 on algal growth

*Thalassiosira rotula* and *S. marinoi* were incubated for 8 days in 48-well plates. Dried CP157 culture extract was dissolved in ESAW, sterile filtered and added to the algal cultures in the following concentrations: 10, 25, 50, 100 mg/ml. Extract prepared from cell-free MB served as negative control. The algae were also co-cultivated with CP157 to evaluate the direct impact of the bacterial cells on algal growth. In brief, CP157 was cultivated in ESAW + 5 mM alanine. The cell pellet was harvested by centrifugation (6,500 rpm, 6 min, 4°C), washed with ESAW and resuspended in ESAW + 5 mM alanine. Bacterial cell density within the algae-containing wells was adjusted to OD_600 *nm*_ = 0.5. As control, untreated algae were incubated in ESAW + 5 mM alanine to exclude an impact of alanine on the growth of the diatoms. Adverse or beneficial effects of treatments on algal growth were identified based on changes in fluorescence at *t*_*x*_ relative to those determined at *t*_0_.

##### Antilarval activity of CP157

Crude extracts of CP157 were applied in a biotoxicity assay modified after Michael et al. (1956) using *A. salina* larvae. Treatments were tested in 12-well plates with each well containing 30 *A. salina* specimens. CP157 crude extracts, cells and MB medium control were prepared and applied as described for treatment of diatoms in the concentrations 10, 25, and 50 mg/ml. Potassium dichromate (50 μg/ml) was used as a positive control for biotoxicity and wells containing untreated larvae served as negative controls. The 12-well plates were incubated at room temperature. Surviving larvae were counted daily under a stereo microscope over a period of 4 days.

#### Production Characteristics of Bioactive Compounds of CP157

##### Production during different growth phases

Production of antimicrobial substances by CP157 was investigated at OD_1__/__4__*max*_, OD_1__/__2__*max*_, OD_3__/__4__*max*_, and twice at OD_*max*_ with an incubation time of 120 h between the last two samplings. To rule out any impact of pigment production on the optical density, at each sampling time point 2 ml culture supernatant were obtained by centrifugation (7,000 rpm, 10 min, 4°C), sterile filtered and the OD_600 *nm*_ was determined. The pH of the culture liquid was monitored using a WTW pH meter 526 (WTW GmbH, Weilheim, Germany). To enable comparison of productivity between sampling time points, the extracted culture volume was adjusted according to the OD (double the volume was extracted at OD_1__/__2__*max*_ as compared to OD_*max*_). All extracts were tested for antagonistic activity against *Paracoccus* sp. CP32. Statistical analyses were performed using R (version 3.6.2) and RStudio (version 1.0.44) (R Core Team, 2020). Results from inhibition assays were tested for normality using the Shapiro-Wilk’s W-test and for homogeneity of variances using Levene’s test. Welch’s *t*-tests were then used for pairwise comparison of means of inhibition zones during different time points of growth (per time point: *n* = 9, *p* = 0.05).

##### Antimicrobial activity in supernatant and cell pellets of CP157 and substrate-dependent production

Substrate-dependent production of bioactive metabolites was investigated by preparing CP157 culture extracts from incubations in ASW + alanine, ASW + proline, and ASW + glucose. Each substrate was supplied in 10 mM concentration. Extracts were tested against CP32 for antagonistic activity.

To determine whether antimicrobial activity is attributed to exometabolites or release of intracellular compounds by cell lysis, extracts prepared from cell-free supernatants and cell pellets of CP157 were checked for biological activity. In brief, 250 ml culture liquid was removed and centrifuged (7,000 rpm, 4°C, 30 min). Supernatants were sterile filtered and incubated with XAD-7 for 2 h. Cell pellets were washed two times with 1x phosphate buffer (PBS, Chazotte, 2012), centrifuged, re-dissolved in ice-cold MeOH and frozen immediately at −80°C for 30 min. Then, pellets were dissolved in water and incubated with XAD-7 for 2 h. The adsorbent was extracted as described above. The resulting extracts were screened for antimicrobial activity. Extracts were prepared from cultures OD_1__/__2__*max*_ to minimize release of metabolites by cell lysis and compared with the results obtained for OD_*max*_.

### Chemical Characterization of Bacterial Crude Extracts

#### Physico-Chemical Stability

The physico-chemical stability of bioactive compounds was tested by exposing CP157 XAD-7 extracts (*aq*) to direct daylight, to 40, 60, 80, and 100°C as well as pH 2, 5, 9, and 12 for 15, 30, 60, and 120 min each. The pH treatment was achieved by re-solubilization of dried culture extract in solvent (ultrapure water) of different pH (adjusted with HCl or NaOH). Extract-free solvent with different pH was used as negative control. The chemical stability of extracted bioactives was further tested by treatment with proteinase K (20 mg/ml) at 37°C for 45 min. Antibacterial activity was tested directly after exposure.

#### Chemical Analysis

Crude extracts from 18 *Paracoccus* spp. were dissolved in MeOH for analysis via LC/MS and in dichloromethane (DCM) for GC/MS (1 mg extract/10 μl solvent). Samples were paper-filtered prior to measurements.

##### Chemical analysis of Paracoccus culture extracts via LC/MS and separation via HPLC

Culture extracts were investigated for their chemical composition with an HPLC system consisting of an Accela autosampler and Accela 1250 pump coupled to an LTQ XL mass spectrometer (Thermo Scientific, Dreieich, Germany) for HPLC/HESI-MS analyses. Heated electrospray ionization (HESI) was used with enhanced scan range of 150–2,000 amu. The gradient program is shown in Supplementary Table 2. Peaks detected in the extracted MB medium control were removed from the dataset. Remaining peaks were filtered to remove background noise and only intensities >10 000 were considered as compound signals for comparison of *Paracoccus* culture extracts.

All measurements were performed with biological triplicates. For visualization and comparison, mean values of signal intensities obtained for each compound were used. For cluster analysis of metabolite profiles, the mean LC/MS signals were normalized by z-scoring and euclidean distances were calculated between profiles of all strains. Distances were clustered using Ward.D2 clustering. Linear models were used to determine relationships between euclidean distance and 16S rRNA gene sequence difference. A PERMANOVA was used to determine the amount of explained variance within the euclidean distances (Benjamini and Hochberg, 1995), adjusted *p*-values ≤ 0.05 were considered to be significant. All analyses on the LC/MS data obtained for XAD-7 and XAD-16 extracts were performed using R (version 4.0.3, R Core Team, 2018) and the vegan package (version 2.5-7, Dixon, 2003).

For separation of extract components, an Agilent ZorbaxEclipse Plus C18 (3.5 μm, 2.1 × 150 mm) column was used at 30°C. Fractions (15/min) were collected using a Dionex Ultimate 3000 HPLC system equipped with a diode array detector and automated fraction collector (Thermo Scientific, Dreieich, Germany). Separation was carried out at room temperature on a Nucleodur C18 HTec column from Machery-Nagel (Düren, Germany). An acetonitrile-water solvent gradient program was used (Supplementary Table 3). All solvents used were of HPLC grade purity. The obtained fractions were tested for antibacterial activity against *Paracoccus* spp. CP32, CP35 and *Arthrobacter* sp. CP30.

##### Identification of AHLs in Paracoccus culture extracts via GC/MS

*Paracoccus* extracts were screened for AHLs via GC/MS. Dimethyl disulfide (DMDS) derivatization for the determination of double-bond positions was carried out by mixing (1:10) 0.24 M iodine-solution and DMDS in DCM and adding 50 μl crude extract. Samples were heated to 50°C for 15 h. Then the mixture was diluted with 200 μl DCM and washed three times with saturated Na_2_S_2_O_3_-solution. The organic phase was dried over Na_2_SO_4_ and directly subjected to GC/MS. Analysis was carried out on a GC 7890A gas chromatograph connected to a 5975C mass-selective detector (Agilent, Santa Clara, CA, United States). Conditions were as follows: carrier gas (He): 1.2 mL min^–1^; injection volume: 1 μl; injector: 250°C; transfer line: 300°C, EI 70 eV. The gas chromatograph was programmed accordingly: 50°C (5 min isothermal), increasing with 5°C min^–1^ to 320°C, and operated in splitless mode. The positions of double-bonds were confirmed by DMDS derivatization as described (Neumann et al., 2013; Ziesche et al., 2019).

## Results

### Phylogenetic Analysis of *Paracoccus* spp.

**FIGURE 1:**
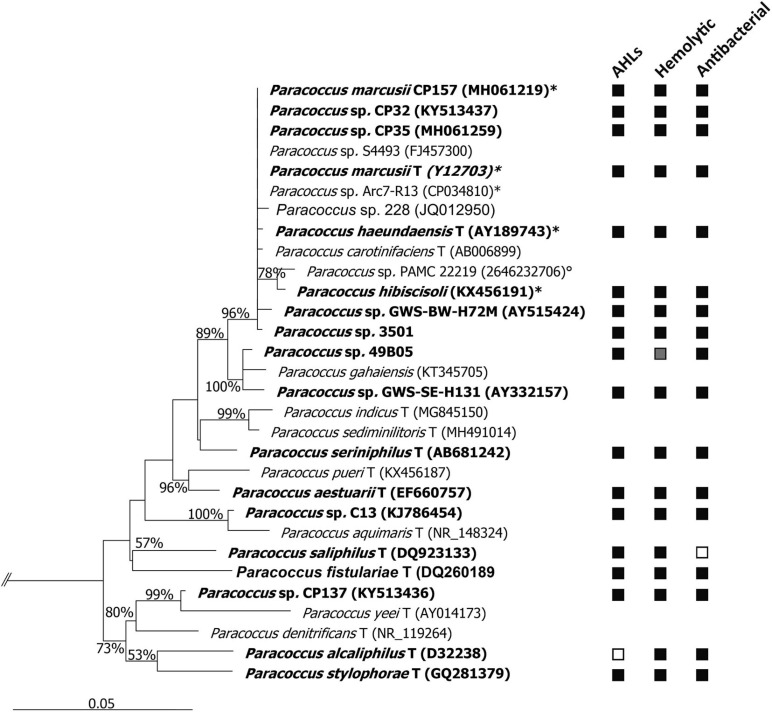
Neighbor-joining tree based on 16S rRNA gene sequence similarity, showing the phylogenetic affiliation of all *Paracoccus* spp. analyzed in this study (in bold). Positive (■) or negative (□) results for secondary metabolites screenings are given behind the respective strain. *Paracoccus* sp. 49B05 did not grow on blood agar plates, hence no result (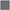) could be recorded. The tree was calculated with sequences ≥1,250 bp. Only bootstrap values ≥50% (derived from 1,000 replicates) at main nodes are shown. Selected sequences related to *Enterobacteriaceae* (AB242910, HG972968, HQ122932, and KF516242) were used as outgroup to define the root of the tree. Root not shown (cut at//). GenBank accession numbers are given in parentheses. ^∗^Strains subjected to *in silico* DDH,° 16S rRNA gene sequence obtained from JGI (https://img.jgi.doe.gov/), T type strain of species.

The phylogenetic distance between the studied strains and our model organism CP157 ranges between 95 and 100% 16S rRNA gene sequence similarity. *A* > 99% sequence identity was detected between CP157, CP32, CP35, GWS-BW-H72M, 3501, DSM 11574T, CGMCC 1.8012, and KACC 18933T, which is reflected by the phylogenetic analysis (Figure 1). *In silico* DDH of the genomes of CP157, *Paracoccus* sp. Arc7-R13, DSM 11574T and CGMCC 1.8012 confirmed a high genetic similarity between these strains (77.1–77.9% identity, Supplementary Table 4), indicating that these organisms belong to one species (Wayne et al., 1987). The type strain of *P. hibiscisoli* KACC 18933T, however, showed only 31.1–31.6% similarity to any of the other four strains. This suggests that KACC 18933T is a different species, despite its 99% identity on 16S rRNA gene level.

### Genome Analysis of CP157

#### General Genome Properties

The complete genome sequence of strain CP157 has a size of 4.08 Mbp. It consists of a 3.07 Mbp chromosome and 22 circular, extrachromosomal elements (ECs), ranging in size between 250 and 1.2 kbp. The GC content of the whole genome is on average 66.44% with pronounced differences between the individual replicons (Supplementary Table 5). The eggNOG mapper assigned COG categories to 3,265 genes. The majority of assigned functions falls into the category *metabolism* (1,266 hits), followed by *information storage and processing* (655 hits) and *cellular processing and signaling* (604 hits), while 740 genes could not be assigned any known function. Eight GIs and eight potential prophage regions were found in the genome of CP157 with partial overlap (Supplementary Tables 6, 7). The GIs are 4.5–80.7 kbp long and cover the chromosomal prophage regions 1, 2 and 4, while prophage 3 is not located within a designated GI. Prophages 5–8 are located on plasmids. The length of prophage regions ranges between 4.5 and 16.4 kbp, covering 7–19 protein coding genes. Of these, only 1–5 proteins were similar to proteins found in the best matching prophage. All prophages were designated *incomplete* or *questionable* due to the low completeness score assigned by PHASTER. No genes for attachment sites were found in any of the prophage regions. Direct pBLAST of known GTA genes against the CP157 genome did not lead to the identification of any GTAs.

#### Genetic Potential of CP157 for Secondary Metabolite Production

Analysis with antiSMASH 5.2 run in strict mode led to the identification of eight biosynthesis gene clusters (BSGCs). While one large hybrid cluster, harboring features of homoserine lactones (HSRs), non-ribosomal peptide synthetases (NRPSs), and polyketide synthases (PKSs), as well as a bacteriocin cluster are located on the 225 kbp EC (pCP157_02), all other clusters were found on the chromosome (Table 1). In the following, BSGCs and other genes potentially involved in secondary metabolite biosynthesis are presented together with results from physiological screenings. All genes presented in the following, are listed in Supplementary Material.

**TABLE 1:** Biosynthesis gene clusters (BSGCs) detected in the genome of *Paracoccus marcusii* CP157 by antiSMASH 5.2 run in strict mode.

Element	Locus (from)	Locus (to)	Size (kbp)	Predicted BSGC type	Most similar known BSGC
cCP157	CP157_00995	CP157_01008	14.4	Siderophore	
	CP157_01012	CP157_01024	10.4	Ectoine	Ectoine (100%)
	CP157_01581	CP157_01601	26.4	Betalactone	
	CP157_02139	CP157_02159	23.6	Terpene	Carotenoid (100%)
	CP157_02177	CP157_02216	41.1	Type 3 PKS	
	CP157_02432	CP157_02457	20.6	Homoserine lactone (HSR)	
pCP157_02	CP157_03436	CP157_03492	74.4	Hybrid (HSR, NRPS, type 1 PKS)	
	CP157_03498	CP157_03507	10.8	Bacteriocin	

*HSR, homoserine lactone; PKS, polyketide synthase; NRPS, non-ribosomal peptide synthetase.*

Thirteen putative hemolysin genes were identified in the CP157 genome (Supplementary Material). Nine are located on the chromosome while the others are encoded on pCP157_02, pCP157_03 and pCP157_06. Three are located within the boundaries of a T3PKS and hybrid BSGCs. All extrachromosomal hemolysin genes (*cya1-4*) show >85% (pBLAST) sequence similarity to the adenylate cyclase *cya* gene of *Bordetella pertussis* 18323 (J7QLC0). In contrast, chromosomal hemolysin genes *cya5-8* are only 27–63% similar to the reference gene of *B. pertussis*. Two other genes found in the genome of CP157 show >30% amino acid sequence similarity with hemolysin genes of *Escherichia coli* J96 (*hlyA*, P09983) and *Rickettsia typhi* ATCC VR-144 (*tlyC*, Q68W10), while two hypothetical hemolysin genes (cCP157_02193, cCP157_02726) remain uncharacterized. pBLAST revealed a generally high similarity of all hemolysin genes to database entries from other *Paracoccus* spp. Hemolytic activity of CP157 was confirmed experimentally using blood agar plates (Figure 1).

Genes for two AHL synthetases were found in the genome of CP157, one on the chromosome, the other on pCP157_02. Both are accompanied by a corresponding transcriptional regulator gene. The extrachromosomal AHL synthetase gene is located within one large hybrid cluster spanning also neighboring NRPS and PKS subclusters. Production of AHLs by CP157 was confirmed experimentally using *A. tumefaciens* as biosensor. In addition, analysis via GC/MS led to the identification of four AHLs in culture extracts of CP157, however, they were not detected by LC/MS due to their low abundance. Mass spectra of the compounds showed ions at *m/z* = 102 and *m/z* = 143, characteristic for AHLs. The major unsaturated AHLs *N*-(9-hexadecenoyl)-homoserine lactone (9-C16:1-AHL) and *N*-(11-ocatadecenoyl)-homoserine lactone (11-C18:1-AHL) were accompanied by minor amounts of their saturated analogs C16:0-AHL and C-18:0-AHL, respectively (Table 2).

**TABLE 2:** Acyl-homoserine lactones (AHLs) detected in *Paracoccus* crude extracts via GC/MS analysis.

Strain		Acyl-homoserine lactone			
ID	(Potential) Species	9-C16:1-AHL	11-C18:1-AHL	C16:0-AHL	C18:0-AHL
CP157	*Paracoccus marcusii*	+/+	+/+	+/+	+/+
CP32	*Paracoccus marcusii*	+/+	+/+	+/+	+/+
CP35	*Paracoccus marcusii*	+/+	+/+	+/+	+/+
GWS-BW-H72M	*Paracoccus marcusii*	−/−	−/−	−/−	−/−
DSM 11574 T	*Paracoccus marcusii*	+/+	+/+	+/+	+/+
3501	*Paracoccus marcusii*	±	±	−/−	−/−
LMG P-21903T	*Paracoccus haeundaensis*	+/+	−/+	−/+	−/+
GWS-SE-H131	*Paracoccus hibiscisoli*	±	±	±	−/−
KACC 18933T	*Paracoccus hibiscisoli*	±	−/−	−/−	−/−
49B05	*Paracoccus liaowanqingii*	±	−/−	−/−	−/−
DSM 14827T	*Paracoccus seriniphilus*	±	−/−	−/−	−/−
CP137	*Paracoccus yeei*	−/+	−/+	−/−	−/−
DSM 19484T	*Paracoccus aestuarii*	−/+	−/−	−/−	−/−
DSM 8512T	*Paracoccus alcaliphilus*	−/−	−/−	−/−	−/−
C13	*Paracoccus aquimaris*	+/+	−/−	−/+	−/−
DSM 18447T	*Paracoccus saliphilus*	−/−	−/−	−/−	−/−
LMG 25392T	*Paracoccus stylophorae*	−/+	−/+	−/+	−/+
KCTC 22803T	*Paracoccus fistulariae*	+/+	+/+	−/−	−/−

*Extracts were prepared with the two resins XAD-7 and XAD-16; the respective results are given in this order 7/16. +, detected; −, not detected. Strains are listed according to their 16S rRNA gene sequence similarity with CP157 from top to bottom. Type strains are marked with T.*

A terpene BSGC consisting of the six genes *crtWZYIBE* was found, encoding the biosynthesis pathway for the carotenoid astaxanthin (MIBiG cluster BGC0000630). Observations from the laboratory confirm the production of an orange pigment by CP157. In addition, Bergen (2018) identified the carotenoid as astaxanthin via HPLC-MS. Interestingly, the genes *crtIYZW* (CP157_02148-02151) were identified as part of the potential GI_05. However, these five genes were only recognized by one of three algorithms incorporated into IslandViewer and form the boundaries of the GI.

Four genes of the biosynthesis pathway for the siderophore petrobactin, *asbABEF* (MIBiG cluster BGC0000942), were detected by antiSMASH (Supplementary Material). However, the two genes *asbCD* that would complete the petrobactin BSGC were not found, neither by antiSMASH nor direct pBLAST. Due to experimental evidence for siderophore production of CP157 (Martine Berger, ICBM, private communication), it can be hypothesized that other genes mediate the encoded functions of *asbCD*, yielding a product similar but not identical to petrobactin. For instance, upstream of *asbAB*, an acyl-carrier protein (CP157_00999) is encoded that may take over the role of the aryl-carrier protein encoded in *asbD* in the petrobactin biosynthesis pathway (Nusca et al., 2012).

Biosynthetic gene clusters potentially involved in bioactivity include PKSs, NRPSs, bacteriocins, and betalactones. Their gene products and cluster organization are often more diverse and hence show lower similarity with known database entries. The bacteriocin cluster found on pCP157_02 consists of ten genes, of which three are upstream of the core biosynthesis gene and oriented in reverse direction. The core biosynthesis gene is uncharacterized, but contains a DUF692, which is characteristic for bacteriocin biosynthesis genes. AntiSMASH identified one large hybrid BSGC on pCP157_02 consisting of the extrachromosomal AHL synthase gene (CP157_03447) mentioned above, several putative PKS and NRPS genes. Most of the PKS and NRPS genes show a 30–60% amino acid sequence similarity to homologous genes in the MIBiG database. A separate PKS cluster was found on cCP157, with a type 3 polyketide synthase as core biosynthesis gene, several response regulators and histidine-kinases. The chromosomal betalactone cluster includes several catabolic genes and a sensor histidine-kinase, but shows no overall correlation with known BSGCs. The similarity to antiSMASH database entries was low (5–28%) and yielded no matches with other *Paracoccus* genomes.

AntiSMASH results obtained for CP157 were compared with those for 50 other *Paracoccus* strains. A total of 303 BSGCs belonging to 15 different cluster types (Supplementary Table 8) were identified by antiSMASH. The chromosomal AHL cluster of CP157 had very high synteny (92–100%) with other *Paracoccus* AHL clusters. BSGCs involved in AHL production were found in all *Paracoccus* strains. While 75% of the compared strains harbored only one AHL cluster, 25% had 2–4 different ones, indicating that *quorum sensing* is a general and important feature of this genus. The next most common cluster types were NRPS (in 63% of the compared *Paracoccus* strains), PKS-NRPS-HSR-hybrid clusters (60%) and bacteriocin (52%). NRPS clusters of 13 *Paracoccus* in the database showed partial (20–42%) similarity with biosynthesis pathways of known siderophores, for instance myxochelin, fusachelin, and vicibactin. In most cases, the similarity extended over 3–4 core or accessory biosynthesis genes (Supplementary Figure 1). The HSR-PKS-NRPS hybrid cluster (on pCP157_02) of CP157, was also found in the genomes of *P. haeundaensis* CCGMCC 1.8012 and *Paracoccus* sp. Arc7-R13 (>95% identity). The same elements found in the hybrid BSGC were identified, however, as individual clusters in the genomes of *P. marcusii* and *P. hibiscisoli* type strains. It can be assumed that their separation on different scaffolds has prevented identification as one large cluster. Comparison of the CP157 bacteriocin cluster with antiSMASH database entries led to matches between all ten genes and bacteriocin BSGCs found in the close relatives *Paracoccus* spp. 228 and Arc7-R13. Interestingly, all other hits in the database show a high synteny as well, but possess only the seven genes that neighbor the core biosynthesis gene in the CP157 gene cluster. Further fairly common BSGCs encode for ectoine (39%), betalactone (29%), T1PKS (27%), terpene (27%, mostly carotenoids), T3PKS (24%), and siderophore (24%). T1PKS and T3PKS do not occur together in most strains, the exceptions being *Paracoccus* sp. BM15, CP157 and its close relative *Paracoccus* sp. PAMC 22219. All BSGCs found in the genomes of CP157, DSM 11574T, CCGMCC 1.8012, and Arc7-R13 were highly similar (80–100%) in organization and size (Supplementary Figure 1). In contrast, the similarity between BSGCs found in the type strain of *P. hibiscisoli* and the clusters detected in the other four strains was much lower (30–60%), which coincides with their lower genome similarity revealed via DDH.

### Bioactivity of CP157 Culture Extracts

#### Broad-Range Antimicrobial Activity of CP157 Culture Extracts

Extracts prepared from CP157 cultures grown in MB showed antagonistic activity toward eight out of ten target strains and inhibited all strains retrieved from the same habitat (Supplementary Table 9). The largest inhibition zones (>3 mm) were observed when CP157 extracts were tested against its close relative CP32 from the same habitat. Not affected were *E. coli* and CP157 itself which reveals self-resistance to the produced substance. Extracts prepared using XAD-7 showed stronger inhibition than those prepared with XAD-16. When tested against *Aquimarina* sp. CP51 and *Staphylococcus* sp. CP100, only the XAD-7 extract showed antimicrobial activity, while the XAD-16 extract did not inhibit growth of the same target strains.

Culture extracts were prepared from CP157 grown with alanine, glucose and proline, substrates found to support growth of CP157 in minimal medium (data not shown). CP157 reached the highest OD_*max*_ (1.12) and antagonistic activity (AU = 1.17 ± 0.28) with alanine, followed by proline (OD_*max*_ = 0.78, AU = 0.56 ± 0.17). Inhibition of extracts after growth with proline was only observed for 2/3 of the biological replicates. For extracts prepared after growth with glucose only one technical replicate showed antagonistic activity, which did not allow calculation of AU. Reproduction of this result led to the conclusion that glucose does not provide a suitable substrate for reproducible production of the antibacterial compound.

#### Effect of CP157 Cultures and Extracts on Growth of Microalgae and *A. salina* Larvae

**FIGURE 2:**
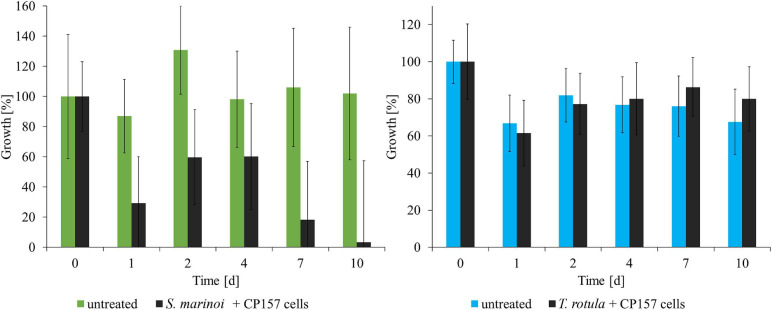
Response of microalgae to co-cultivation with CP157 (black) and comparison with untreated cultures of *Skeletonema marinoi* (green) or *Thalassiosira rotula* (blue). Growth was determined by measuring relative fluorescence over time. Changes are expressed as percentage relative to the fluorescence measured at *t*_0_. Values are means ± standard deviation (*n* = 3).

*Thalassiosira rotula* did not show any response to the presence of CP157 cells (Figure 2). Incubation with 10–50 mg/ml CP157 culture extract led to an increase in relative fluorescence (+18–27%), which indicates a positive effect of CP157 extracts on growth of *T. rotula* (Supplementary Figure 2). Treatment of *T. rotula* with MB extract (medium control) did not show any pronounced effects when added at 100, 25, and 10 mg/ml. Addition of 50 mg/ml MB extract, however, led to an increase in fluorescence (+2–12%) (Supplementary Figure 2). Hence, we assume a slightly positive impact of MB components on the growth of *T. rotula*, which is even more pronounced after addition of CP157 extract.

*Skeletonema marinoi* showed a strong negative response to the addition of CP157 cells (Figure 2). Within the first 24 h relative fluorescence decreased to 29% of the initial value, and at the end of the experiment only 3% of the fluorescence measured at t_0_ were retained. Growth of *S. marinoi* was also negatively affected by any type of extract (MB/culture), possibly related to MB medium components (Supplementary Figure 2). Clear differences between treatment with CP157 extract or MB extract were observed only at the lowest concentration (10 mg/ml). *S. marinoi* cultures treated with 10 mg/ml CP157 extract showed a strong decrease in relative fluorescence (−46%) within the first 24 h (Supplementary Figure 2). Subsequently, relative fluorescence continued to decrease until only 19% fluorescence was retained at the end of the experiment. In contrast, *S. marinoi* cultures treated with 10 mg/ml MB extract showed a strong increase of relative fluorescence within the first 2 days (+79%). This was followed by a continuous decrease of measured values. At the end of the experiment 73% fluorescence was retained, indicating that adverse effects caused by MB components are most pronounced at high concentrations thus concealing any other effects possibly caused by CP157 extracts. It should be noted, that chain formation of microalgae and the resulting uneven distribution in the wells led to high variations between measured values obtained for biological or technical replicates.

**FIGURE 3:**
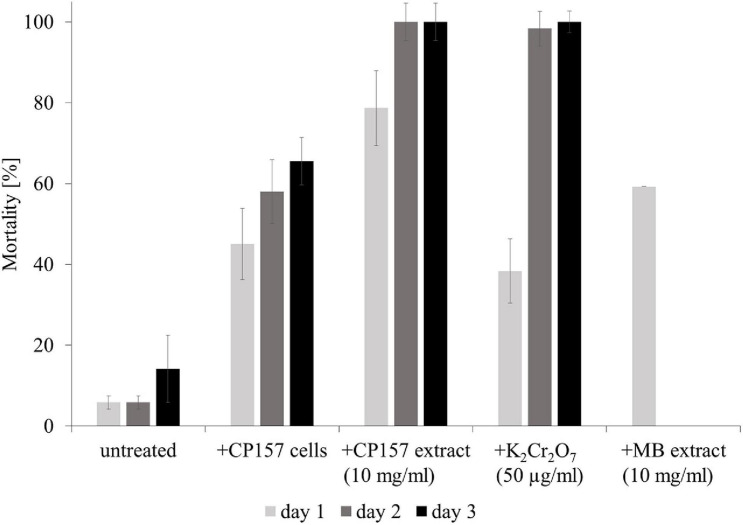
Mortality of *Artemia salina* larvae when untreated, exposed to CP157 cells, CP157 culture extract or MB extract. For the latter two, the effects of the lowest tested dose (10 mg/ml) are shown exemplarily. K_2_Cr_2_O_7_ served as a positive control for biotoxicity. Values are means ± standard deviation (*n* = 3).

The effect of CP157 cells and culture extracts on *A. salina* larvae was observed for 4 days. Natural mortality of larvae in untreated wells increased greatly after day 3 due to starvation and accumulation of waste products. Consequently, only effects of tested treatments observed within the first 3 days after exposure are presented. After 1 day of incubation, *A. salina* exposed to CP157 cells (in ESAW) showed a 44% higher mortality than in untreated wells containing the same medium (Figure 3). At day 3 mortality in wells containing *A. salina* + CP157 cells had increased to 66% opposed to 14% in untreated control wells. Treatment of *A. salina* with CP157 culture extracts caused a mortality of 80–95% in the first 24 h (Figure 3). No living larvae were observed after day 1. Even though treatment of *A. salina* with MB extract (medium control) also resulted in increased mortality (40–60%, day 1), the effect was clearly less pronounced compared to CP157 culture extracts (Supplementary Figure 3). Unfortunately, wells containing *A. salina* + MB extract were always contaminated with bacteria after >24 h, leading to 100% mortality. Under the tested conditions, *A. salina* cannot be grown aseptically and addition of MB extract provides sufficient nutrients to support growth of diverse bacterial contaminants.

#### Production of Extracellular Antibacterial Metabolites Throughout Different Growth Phases

**FIGURE 4:**
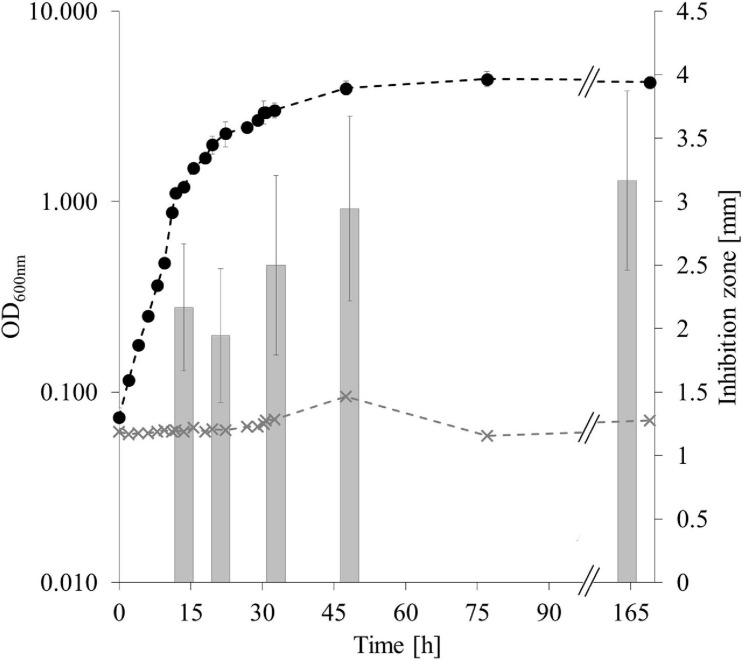
Antagonistic activity of CP157 throughout different growth phases. Growth of CP157 (black dashed line) and medium control (gray dashed line) measured at OD_600__*nm*_. Gray bars represent the antagonistic activity in mm at the sampling points. Samples were taken at OD_600__*nm*_ = 1, 2, 3, 4 (*t*_1_**_–_**_4_) and in the late stationary phase (*t*_5_). For means of visualization, the X-axis was shortened at //, without cutting off any measurements. Values are given as means ± standard deviation (*n*_*OD*_ = 3, *n*_*inhibition*_ = 9).

Extraction of secondary metabolites in a constant cell density-to-volume ratio showed that antagonistic activity of CP157 at OD_1__/__4__*max*_ (*t*_1_), OD_1__/__2__*max*_ (*t*_2_), OD_3__/__4__*max*_ (*t*_3_), and OD_*max*_ (*t*_4_,_5_) does not change significantly (*p* = 0.05, *n* = 9) over time (Figure 4). Consequently, accumulation of antagonistic substances, indicated by inhibition zones increasing over time, as observed in previous experiments, may be due to the rising number of producer cells instead. Spectrophotometric examination of the cell-free supernatant confirmed that pigment production had no effect on the optical density, even though the culture broth visibly changed color. An increase in pH from 6.97 (*t*_1_) to 8.34 (*t*_5_) was observed over time.

Separate extraction of cell pellets and cell-free supernatant of CP157 cultures and a subsequent inhibition test revealed antagonistic activity toward CP32 of both extract types to a similar extent, confirming the presence of antibacterial metabolites within the cells as well as in the supernatant.

#### Thermo- and pH-Stability of CP157 Antimicrobials

**FIGURE 5:**
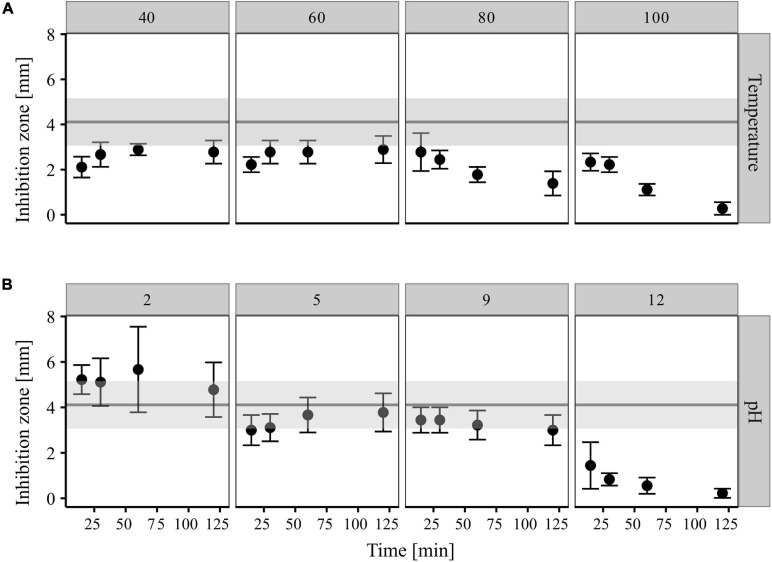
Impact of temperature **(A)** and pH **(B)** treatments on antagonistic activity of CP157 cultures extracts (XAD-7, 50 mg/ml, *aq*). Different exposure times are given on the X-axis. Range of inhibition zones caused by untreated culture extract (control) are represented by the gray horizontal bar. Values are means (95% confidence interval, *n* = 9). The figure was created using the tidyverse package in R (Wickham et al., 2019).

Exposure of CP157 crude extract to 40, 60, 80, and 100°C for 15, 30, 60, and 120 min led to a reduction in antimicrobial activity in all cases (Figure 5A). In comparison to the untreated culture extract, the inhibition zones around the heat-treated samples were on average 46% smaller. A pronounced effect on the antimicrobial activity was observed when the extract was heated for 60 and 120 min to 80 or 100°C. In this case, the resulting inhibition zones were 57–93% smaller than those produced by untreated culture extract (Figure 5A). Treatment of the CP157 culture extracts with pH 2 seemed to have a positive effect on the antimicrobial activity, i.e., inhibition zones of culture extracts adjusted to pH 2 were on average 27% larger than those caused by untreated extract (Figure 5B). No inhibition zones formed around pH 2 controls (extract-free solvent). The opposite effect was observed for samples treated with pH 12. Starting with a 65% reduction of inhibition zones after 15 min of exposure, antagonistic activity of the extract gradually decreased with exposure time with only 5% activity remaining after 120 min (Figure 5B). Intermediate pH values (pH = 5; pH = 9) resulted in a minor decrease of bioactivity which ranged between 8 and 27%. Incubation of CP157 culture extract with proteinase K resulted in a 38% loss of biological activity (data not shown).

### Bioactive Metabolite Production Found in Other *Paracoccus* spp.

To distinguish the strain-specific metabolic potential of CP157 from general characteristics of *Paracoccus* spp., 17 additional strains were screened for production of secondary metabolites. A summary of the screening results is given in Figure 1.

#### Production of Bioactive Metabolites by Different *Paracoccus* spp.

The four different AHLs identified via GC/MS in extracts of CP157 were also detected in other *Paracoccus* strains. Most prevalent was the 9-C16:1-AHL which was found in 14 strains, followed by 11-C18:1-AHL (10 strains), C16:0-AHL (8 strains), and C18:0-AHL (6 strains) (Table 2). All *P. marcusii* strains isolated from CP, the type strains of *P. marcusii* and *P. haeundaensis*, which all belong to the same species (Figure 1), as well as the type strain of *P. stylophorae* produced all four AHL derivatives, while most other strains produced only the unsaturated variants. No AHLs were found in extracts of *P. alcaliphilus* and *P. saliphilus*. All *Paracoccus* spp. except *P. alcaliphilus* were tested positive for AHL production using the biosensor *A. tumefaciens*. The discrepancy, which arises from the combined results for *P. saliphilus*, is most likely due to low concentrations of AHLs in methanolic culture extracts.

Hemolytic activity was observed after 96 h on blood agar plates for CP35, C13, 3501, *P. aestuarii*, *P. haeundaensis*, *P. hibiscisoli*, *P. marcusii*, *P. fistulariae*, and *P. alcaliphilus*. In the following 96 h, hemolysis was also observed on plates inoculated with CP137, GWS-SE-H131, GWS-BW-H72M, *P. saliphilus*, and *P. seriniphilus*. After a total incubation time of 14 days, hemolysis was also apparent for the remaining *Paracoccus* strains CP32 and *P. stylophorae*. *Paracoccus* sp. 49B05 was the only strain, which did not grow on blood agar plates, hence its ability to produce hemolysins could not be evaluated with this test (Figure 1).

All *Paracoccus* culture extracts except that of *P. saliphilus* showed antimicrobial activity against *Paracoccus* spp. CP32 and CP35. In addition, growth inhibition of *Arthrobacter* sp. CP30 and *Aquimarina* sp. CP51 was observed for 11 and 13 *Paracoccus* strains, respectively (Supplementary Table 9). Interestingly, *P. marcusii* CP32 was susceptible to its own culture extracts, contrasting with findings for CP157. Subsequent cross-testing of CP157 and the five type strains of *P. haeundaensis*, *P. marcusii*, *P. alcaliphilus*, *P. seriniphilus*, and *P. stylophorae* revealed self-resistance in some strains (Supplementary Figure 4 and Supplementary Table 9). While CP157, *P. seriniphilus* and *P. stylophorae* were not inhibited by their own culture extracts, *P. marcusii* and *P. haeundaensis* were strongly inhibited by their extracts. Overall, strongest inhibitions and broadest inhibition ranges were found for the type strains *P. haeundaensis* and *P. stylophorae*, followed by the strains CP157 and C13 (Supplementary Table 9).

#### Chemical Analysis of *Paracoccus* spp. Crude Extracts Reveals Presence of Several New Candidate Antimicrobial Compounds

After curation, crude extracts from 18 *Paracoccus* spp. contained a total of 2,589 and 2,386 different peaks in XAD-7 and XAD-16 samples respectively. The number of corrected peaks found per strain in XAD-7 and XAD-16 extracts ranged between 898–381 and 1,013–535, respectively (Supplementary Table 10). For 15 strains more peaks were recorded in XAD-16 than XAD-7 extracts. It has to be mentioned that the true number of compounds is clearly lower than the number of distinguished peaks in each extract due to formation of adducts and additional peak formation for isotopes.

**FIGURE 6:**
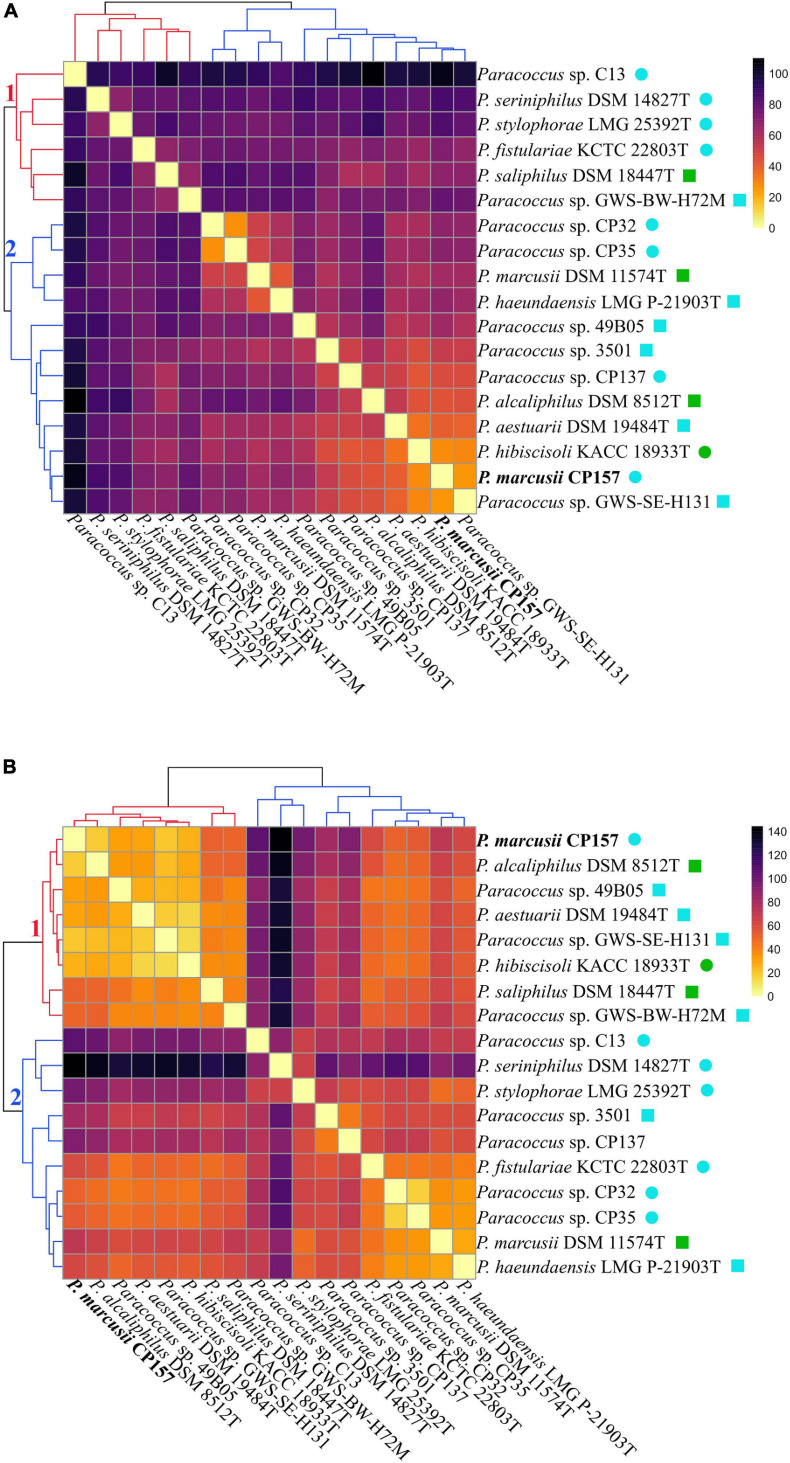
*Paracoccus* culture extracts prepared with **(A)** XAD-7 or **(B)** XAD-16 are clustered according to their similarity (decreasing euclidian similarity from bright to dark) based on their composition (presence/absence of compounds) and the intensity of individual signals. The branches of the two major clusters identified within each data set are numbered and colored in red and blue. Compounds detected in MB extracts were removed and mean values of biological triplicates were normalized by z-scoring prior to plotting. Habitats of the respective strains are indicated as follows: 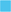 marine + free-living, 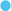 marine + symbiotic, 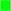 terrestrial + free-living, 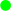 terrestrial + symbiotic.

Cluster analysis based on extract composition and signal intensity revealed that *Paracoccus* extracts prepared with either adsorbent form two distinct clusters (Figure 6, clusters designated 1 and 2). Even though the distribution of strains among the clusters is not identical for the two datasets, several subclusters are similarly arranged. In both cases, extracts from C13, DSM 14827T, and LMG 25392T show high euclidean distances to all other extracts and are moderately to highly dissimilar to each other. Extracts of the closely related type strains of *P. marcusii* DSM 11574T and *P. haeundaensis* LMG P-21903T and of their close relatives CP32 and CP35 are grouped together with low distances. Extracts of the latter two strains cluster very closely together, which indicates that their metabolite profiles are very similar. Interestingly, extracts from CP157 do not belong to this group, but cluster with DSM 8512T, DSM 19484T, KACC 18933T, and GWS-SE-H131. Extracts in this CP157 subcluster show moderate to high similarity to each other (indicated by low euclidean distances), especially the closely related strains CP157, GWS-SE-H131 and the type strain of *P. hibiscisoli* KACC18933T. In the XAD-16 dataset the CP157 subcluster further includes extracts from its close relative 49B05. In both datasets extracts from *Paracoccus* spp. CP137 and 3501 do not fall within a distinct cluster but have quite high distances to all other extracts. In the XAD-16 dataset no significant correlation between euclidean distance of culture extracts and the phylogenetic distance of their producers was observed, while a weak correlation was found in the XAD-7 dataset (Supplementary Figure 5). This is most clearly illustrated in the case of close relatives of CP157 which fall into several different subclusters in either of the two datasets. Moreover, no significant correlation between similarity of extracts and the habitats of their producers was observed.

Separation of CP157 crude extracts via HPLC yielded 13 fractions out of which nine were tested positive for antimicrobial activity against at least one target strain. The bioactivity patterns and extension of inhibition zones varied between the target strains with CP35 being most susceptible (Supplementary Table 11). The presence of inactive fractions between active ones allows the assumption that CP157 crude extracts contain several antibacterial compounds. Further separation of bioactive fractions yielded 243 subfractions out of which 51 were tested positive for antibacterial activity. Out of those, 27 subfractions produced inhibition zones in at least two technical replicates and were thus considered as suitable candidates for downstream analysis (data not shown). The low amount of biological material, however, did not suffice to identify an antibacterial compound. Identification of such compounds requires large scale production to isolate enough material to perform NMR-analysis, which is planned for the future.

## Discussion

### High Potential for Production of Bioactive Metabolites Found in CP157 and Other *Paracoccus* spp.

Within this study, we detected inter-kingdom antagonistic activity of our model organism CP157 against a diatom and a crustacean. Furthermore, antibacterial activity of CP157 and 16 other *Paracoccus* spp. was observed. Bioactivity of the here tested *Paracoccus* strains extends from antibiosis over signaling to hemolysis, of which the latter has so far not been reported for members of this genus. Corresponding genes found in the CP157 genome are similar (>40%) to those of α-hemolysin (*hlyA*) and adenylate-cyclase toxin (*cyaA*) of pathogenic *E. coli* and *Bordetella pertussis*, respectively (Gardiner et al., 2017). In both cases, the gene products are known to possess cytotoxic activity toward a broad range of eukaryotic cell types (Ludwig and Goebel, 2006). These products induce pore formation causing leakage of nutrients from the target cell, which can contribute to survival of the producing bacterium in harsh environments thus increasing the number of potential habitats available to CP157 for colonization (Mogrovejo-Arias et al., 2020).

Though culture extracts had a broad-range antibacterial activity, the effect on closely related strains from the same habitat was the strongest. This is a characteristic of bacteriocins, which are low mass weight, protein-like compounds of variable chemical character (Morton et al., 2015). They have a multitude of functions, but receive most attention for their antimicrobial properties (Azevedo et al., 2015). We found a bacteriocin BSGC with the characteristic DUF692 (Jackson et al., 2018) in the genome of CP157. In addition, bacteriocin clusters are among the three most common BSGCs of *Paracoccus* spp. However, (core) biosynthesis genes could not be associated with any known bacteriocin clusters. Consequently, we assume that CP157 produces a so far unknown bacteriocin-like bioactive compound.

Further, PKS and NRPS clusters are not only encoded in the genome of CP157 but are the most abundant BSGCs found in *Paracoccus* spp. Products from PKS and NRPS clusters are among the most prominent bioactive and pharmaceutically relevant natural compounds (Böhringer et al., 2017). Their activities range from antibacterial and iron scavenging to the biosynthesis of cell wall components or surfactants (Lautru and Challis, 2004; Desriac et al., 2013). Our analysis indicates that in several cases *Paracoccus* NRPS clusters are more likely involved in biosynthesis of siderophores than production of antimicrobials. This theory is further supported by findings from Wang et al. (2014), who assigned the synthesis of individual parabactin moieties in *P. denitrificans* to NRPS. Especially type 1 PKS are, however, known to directly synthesize antimicrobial compounds such as macrolides (Le et al., 2014), type 3 PKS often provide the building blocks for subsequent modification and tailoring by T1PKS or NRPS (Katsuyama and Ohnishi, 2012). Differences observed between *Paracoccus* spp. in their range of antibacterial activity, might possibly trace back to such downstream modification of gene products or synthesis from different substrates. For example T3PKS and especially the adenylation domains of NRPSs are known to accept various substrates, hence producing multiple structural analogs (Lautru and Challis, 2004; Hug et al., 2019). Both leads to the synthesis of strain-specific compounds that differ in their mode of action and thus their efficacy toward bacterial targets (Hug et al., 2019). de Bruijn et al. (2015) sequenced and compared the genomes of five *Lysobacter* spp. (Gammaproteobacteria) with experimental evidence for antimicrobial activity spectra and production of extracellular, lytic enzymes. Similarly to our comparison of *Paracoccus* spp. they found so far unrecognized potential for bioactivity in all tested strains, but distinct differences in the extent of antagonistic activity. Taking into account the different isolation sources of the here tested strains, “customized” bioactive compounds could be the result of adaptation to new environmental conditions (Guerrero-Garzón et al., 2020). This is further supported considering the diverse metabolic profiles found in *Paracoccus* culture extracts (Figure 6). Even though no significant correlation between extract composition and producer habitat could be found, we speculate that highly different and thus rather unique culture extracts of host-associated strains such as C13 or DSM 14827T hint to habitat-specific metabolite production. Future in-depth analysis and comparison of more *Paracoccus* genomes with regard to PKS/NRPS BSGCs and their gene products will help validating this assumption.

In confined habitats characterized by bacterial communities with high species richness such as biofilms, strong selective pressure favors the diversification of metabolic products (Zheng et al., 2019). The low similarity of detected BSGCs with known bacteriocin or NRPS/PKS clusters suggests that their gene products are likely to be novel compounds (Zheng et al., 2019). To the best of our knowledge, there are no described bacteriocins, bioactive polyketides, or ribosomal peptides of *Paracoccus* spp. even though PKS and NRPS clusters have been found in several members of the genus (Graça et al., 2015; Machado et al., 2015). Individual records report bacteriocin or antimicrobial polyketide production in members of the *Roseobacter* group, for instance *Ruegeria*, *Pseudovibrio*, *Sulfitobacter*, or *Roseovarius* spp. (Raimundo et al., 2018; Karimi et al., 2019), but without characterization of the compound chemistry. One of the rare, described examples is that of indigoidine, a NRPS product of *Leisingera* sp. Y41 that shows antagonistic activity toward *Vibrio fischeri* (Gromek et al., 2016). Our comparisons thus indicate a so far unrecognized potential of *Paracoccus* spp. for biosynthesis of diverse bioactive compounds.

### CP157 Produces Multiple Small Antibacterial Compounds

Physico-chemical characterization of crude extracts of CP157 showed that the active compounds are rather polar, resist proteinase K treatment and acidic pH, but slowly degrade over time when exposed to heat and alkaline pH. As stated above, analysis of the CP157 genome indicates that the strain might produce a bacteriocin. Due to their proteinaceous nature, bacteriocins are usually sensitive to heat and proteolytic enzymes. But specialized chemical structures such as salt bridges and hydrogen bonds increase resistance of bacteriocins to unfavorable conditions (Ansari et al., 2018). Based on our results, we assume that a bacteriocin-like compound produced by CP157 may be equipped with such modifications, which, to a certain degree, protect it from degradation by heat or changes in pH.

Most bacteriocins described for Gram-negative bacteria are larger than 20 kDa and only few exceptions within the Gammaproteobacteria such as microcins or colicins are smaller than 10 kDa (Papagianni and Anastasiadou, 2009). Considering the detection limit of our instrumentation to substances <2 kDa, we assume that none of the candidate compounds identified in the culture extract of CP157 represents a bacteriocin, but that they correspond to other, smaller bioactive molecules. Hence we conclude that multiple small antibacterial compounds are produced by CP157 in addition to the presumed bacteriocin. This assumption is further supported by identification of multiple bioactive fractions in CP157 culture extracts. The presence of several chemically distinct, bioactive compounds would also explain the retained inhibition of target strains after heating. Thermal degradation of the bacteriocin would lead to the smaller inhibition zones, while antimicrobial activity of other, heat-stable compounds remains. Based on the identified BSGCs, we assume that further antimicrobials are products of PKS and NRPS or hybrids thereof. Timmermans et al. (2017) summarized information on marine bioactive metabolites, mentioning several examples of PKS-NRPS hybrids such as thalassospiramides and didemnines as products of Alphaproteobacteria. Among the described polyketides are several compounds with masses <1 kDa. Multiple bioactivities are reported for polyketides and NRPs including antibacterial, antifungal or anticancer activities (Desriac et al., 2013). Our attempts to isolate and characterize chemical species from *Paracoccus* culture extracts were limited by low amounts of obtained biological material and the detection range of our instrumentation. Development of improved extraction and isolation procedures for instance via modification of culture conditions and upscaling, to enable the implication of nuclear magnetic resonance spectroscopy, would be required for further analysis.

### Ecological Niche of CP157 and Role of Secondary Metabolites in Adaptation

With 22 ECs, CP157 currently has the genome with the highest number of ECs documented for members of the *Rhodobacteraceae*. This high number of ECs is a proof for genetic flexibility and might provide the basis for fast adaptation to new environmental conditions (Frank et al., 2015). The genetic components required for successful adaptation are often obtained via horizontal gene transfer from an existing bacterial community (Guerrero-Garzón et al., 2020). The numerous ECs likely provide a competitive advantage for CP157, for instance due to additional BSGCs on plasmid pCP157_02.

Based on our results, we assume that CP157 and possibly other *Paracoccus* spp. investigated in this study are opportunistic pathogens to their hosts. This is supported considering that CP157 is ubiquitous on the carapace of diseased *C. pagurus* specimens, but occurs in strongly elevated abundances in black spots (Bergen, 2018). So far, an opportunistic lifestyle of *Paracoccus* spp. has only been described for strains of *Paracoccus yeei*, contributing to wound infection in humans (Lasek et al., 2018). In a state prior to disease development, CP157 may protect its host from settlement of larvae and other pathogens, as has been demonstrated for another member of the *Rhodobacteraceae*, *Phaeobacter inhibens* 2.10, a symbiont of the macroalga *Ulva australis* (Rao et al., 2007). The type of interactions between bacteria and their host organism is determined by multiple environmental factors and exchanged metabolic products. Bacterial symbionts may switch from mutualistic to pathogenic when the external parameters change, as described for *P. inhibens*, producing algicides, once the aging algal host leaks increasing amounts of *p*-coumaric acid (Seyedsayamdost et al., 2011). In *Vibrio corallilyticus*, a coral pathogen, presence of chitin leads to changes in its exometabolome and an increase in secondary metabolite production (Giubergia et al., 2016). In its natural environment, CP157 occurs together with several *Aquimarina* spp. that are particularly abundant in diseased areas on the *C. pagurus* carapace (Kraemer et al., 2020). *Aquimarina* spp. are known chitin-degraders and presumed to play a role in disease progression or even development (Xu et al., 2015; Quinn et al., 2017). We assume that CP157, which is not able to degrade chitin or its monomer *N*-acetylglucosamine itself, benefits from the progressing penetration of the exoskeleton by *Aquimarina* spp. Our hypothesis is supported by a study from Smolowitz et al. (2005), describing the stages of progressing epizooic shell disease in the American lobster, *Homarus americanus*. Initial physical injury is followed by microbial colonization and degradation by chitinolytic bacteria. This is largely accompanied by non-chitinolytic bacteria that colonize spaces in the crystal lattice of chitin, where they feed on proteins and amino acid-rich substrates. This scenario fits particularly well, taking into account that amino acids enable growth of CP157 and production of bioactive metabolites when supplied as single substrates. Considering the growth inhibition of *Aquimarina* sp. CP51 by CP157 culture extracts, we hypothesize that secondary metabolite production of CP157 might be influenced, similarly to *V. corallilyticus*, by elevated concentrations of chitin, its degradation products or the increasing availability of amino acids that may result from abrasion or microbial degradation of the cuticle. Co-cultivation experiments of CP157 with *Aquimarina* strains from the same habitat, or the evaluation of the impact of chitin on CP157 may provide further insight into the ecological role of CP157 secondary metabolites and the interactions with its neighbors.

With regard to bacteria-bacteria interactions, we assume that secondary metabolites of CP157 have a strong impact on the composition of the epibiotic community of *C. pagurus*. Via production of its diverse bioactive compounds, CP157 may prevent or enhance the settlement and development of specific community members in its immediate environment. Exemplarily, Rao et al. (2007) demonstrated that AHLs produced by *Phaeobacter inhibens* 2.10 actively select for specific organisms to colonize *U. australis*. In addition, they showed, that *P. inhibens* 2.10 is able to prevent settlement of other bacteria even at low cell densities. We have observed such selective antibiosis in the inhibition patterns of CP157. The strong antibacterial activity of CP157 to closely related strains combined with self-resistance may thus provide a competitive advantage over occupants of the same ecological niche (Egan et al., 2014; Mullis et al., 2019). Simultaneously, settlement of “useful” neighbors that provide metabolic products as substrates, or a mature biofilm matrix may be favored by attracting them via signaling compounds (Giubergia et al., 2016; Wolter, 2019). Interestingly, antimicrobial activity of another *Phaeobacter* strain (*P. gallaeciensis* BS107) was most pronounced when kept in co-culture with *Vibrio anguillarum* 408 (Ruiz-Ponte et al., 1999). Assuming that CP157 is both emitter and recipient of QS signals, CP157 may respond similarly to environmental factors and microbial cues, either by increased or decreased production of bioactive metabolites.

We have demonstrated that the degree of antibiotic activity in CP157 culture extracts does not increase significantly with incubation time. In a closed system this may result from feedback repression once a certain threshold concentration is reached. This is supported, considering the stability of CP157 culture extracts under the tested conditions, making it more likely that bioactive compounds are not degraded, but that production has ceased. To resolve questions regarding biosynthesis and production kinetics, identification and isolation of pure substances is required. This would further enable investigation of dose-dependent interactions between CP157 secondary metabolites and other microbial community members or host organisms to determine their ecological role.

## Data Availability Statement

The datasets presented in this study can be found in online repositories. The names of the repository/repositories and accession number(s) can be found below: https://www.ncbi.nlm.nih.gov/genbank/, CP065892–CP065914; https://www.ncbi.nlm.nih.gov/, PRJNA693451.

## Author Contributions

JL and TB were the main contributors to conception of the study. JL, A-CK, and JH carried out the experimental work. BB, HF, and CS sequenced, assembled, and annotated the genome of *Paracoccus marcusii* CP157. LD performed the statistical analysis. JL mainly evaluated the collected data, and wrote the initial manuscript that was improved by HF, SS, and TB revisions. All authors read and approved the final manuscript.

## Conflict of Interest

The authors BB, CS, and HF were employed by the non-profit research institution DSMZ. The remaining authors declare that the research was conducted in the absence of any commercial or financial relationships that could be construed as a potential conflict of interest.

## Footnotes

1 https://www.dsmz.de/microorganisms/medium/pdf/DSMZ_Medium772.pdf

2 https://github.com/boykebunk/genomefinish

3 https://www.ncbi.nlm.nih.gov/

4 http://ggdc.dsmz.de/ggdc.php#
