# Supplementary material for: High Potential for Secondary Metabolite Production of Paracoccus marcusii CP157, Isolated From the Crustacean Cancer pagurus
Source: Front Microbiol. 2021 Jun 28;12:688754. doi: 10.3389/fmicb.2021.688754 (PMC8273931; doi:10.3389/fmicb.2021.688754)
Supplement: Supplementary file 1 [file Data_Sheet_1.pdf]

1 Supplementary Material

1.1 Organisms used in this study

Supplement T 1: Organisms screened for production of bioactive metabolites (producers) or served as indicators for bioactivity of *Paracoccus* culture extracts (targets). All *Paracoccus* spp. obtained from culture collections are type strains of the species (T). Algal strains were grown axenically and *Artemia salina* larvae were purchased as a ready-to-use mixture including salts and microalgal food particles. Isolation sources on *C. pagurus* carapace: not affected (NA) by shell disease or black spot (BS). °closest relative of environmental strains determined via nBLAST (blast.ncbi.nlm.nih.gov/Blast.cgi) of the 16S rRNA gene sequence; n.a. not available

Organism					Background			Reference
**Domain**	**Class**	**Genus**	**Species°**	**Strain**	**Habitat/Host**	**Provider**	**Use**	
Bacteria								
	α-Proteobacteria	*Paracoccus*	*aestuarii*	DSM 19484T	Tidal flat sediment	DSMZ	Producer	(Roh *et al.* 2009)
	α-Proteobacteria	*Paracoccus*	*alcaliphilus*	DSM 8512T	Soil	DSMZ	Producer	(URAKAMI *et al.* 1989)
	α-Proteobacteria	*Paracoccus*	*aquimaris*	C13	*Fucus spiralis*	Environmental	Producer	(Dogs *et al.* 2017)
	α-Proteobacteria	*Paracoccus*	*fistulariae*	KCTC 22803T	*Fistularia commersonii*	CCUG	Producer	(Kim *et al.* 2010)
	α-Proteobacteria	*Paracoccus*	*haeundaensis*	LMG P-21903T	Sea water	BCCM/LMBP	Producer	(Lee *et al.* 2004)
	α-Proteobacteria	*Paracoccus*	*hibiscisoli*	KACC 18933T	*Hibiscus syriacus*	KACC	Producer	(Lin *et al.* 2017)
	α-Proteobacteria	*Paracoccus*	*hibiscisoli*	GWS-SE-H131	Marine sediment	Environmental	Producer	(Stevens *et al.* 2005)
	α-Proteobacteria	*Paracoccus*	*liaowanqingii*	49B05	Sea water	Environmental*	Producer	(Fu *et al.* 2019)
	α-Proteobacteria	*Paracoccus*	*marcusii*	DSM 11574T	Airborne	DSMZ	Producer	(Harker *et al.* 1998)
	α-Proteobacteria	*Paracoccus*	*marcusii*	3501	Sea water	Environmental*	Producer	(Fu *et al.* 2019)
	α-Proteobacteria	*Paracoccus*	*marcusii*	CP157	*Cancer pagurus*, BS	Environmental	Producer	This study
	α-Proteobacteria	*Paracoccus*	*marcusii*	CP32	*Cancer pagurus*, NA	Environmental	Producer	This study
	α-Proteobacteria	*Paracoccus*	*marcusii*	CP35	*Cancer pagurus*, NA	Environmental	Producer	This study
	α-Proteobacteria	*Paracoccus*	*marcusii*	GWS-BW-H72M	Sea water	Environmental	Producer	(Stevens *et al.* 2005)
	α-Proteobacteria	*Paracoccus*	*saliphilus*	DSM 18447T	Saline-alkali soil	DSMZ	Producer	(Wang *et al.* 2009)
	α-Proteobacteria	*Paracoccus*	*seriniphilus*	DSM 14827T	*Bugula plumosa*	DSMZ	Producer	(Pukall *et al.* 2003)
	α-Proteobacteria	*Paracoccus*	*stylophorae*	LMG 25392T	*Stylophora pistillata*	BCCM/LMBP	Producer	(Sheu *et al.* 2011)
	α-Proteobacteria	*Paracoccus*	*yeei*	CP137	*Cancer pagurus*, NA	Environmental	Producer	This study
	γ-Proteobacteria	*Escherichia*	*coli*	DSM 613	n.a.	DSMZ	Target	(Daegelen *et al.* 2009)
	γ-Proteobacteria	*Raoultella*	*planticola*	DSM 3069T	Radish root	DSMZ	Target	(Bagley *et al.* 1981)
	γ-Proteobacteria	*Pseudomonas*	*stutzeri*	DSM 5190T	Spinal fluid	DSMZ	Target	(Burri and Stutzer 1895)

	Organism				Background			Reference
**Domain**	**Class**	**Genus**	**Species°**	**Strain**	**Habitat/Host**	**Provider**	**Use**	
	Actinobacteria	*Arthrobacter*	*busseii*	CP30	*Cancer pagurus*, NA	Environmental	Target	This study
	Flavobacteriia	*Aquimarina*	*muelleri*	CP51	*Cancer pagurus*, BS	Environmental	Target	This study
	Flavobacteriia	*Tenacibaculum*	*gallaicum*	CP14	*Cancer pagurus*, BS	Environmental	Target	This study
	Bacilli	*Staphylococcus*	*equorum*	CP100	*Cancer pagurus*, NA	Environmental	Target	This study
	Bacilli	*Bacillus*	*subtilis*	SMS4	Sea water	Environmental**	Target	This study
Eukaryota								
	Branchiopoda	*Artemia*	*salina*	n.a.	n.a.	JBL GmbH & Co. KG	Target	n.a.
	Coscinodiscophyceae	*Skeletonema*	*marinoi*	CCMP 1332	Sea water	CCMP	Target	n.a.
	Coscinodiscophyceae	*Thalassiosira*	*rotula*	CCMP 1647	Sea water	CCMP	Target	n.a.

*provided by Andrew Lang, MUN, St. John’s, Canada
**provided by Marion Pohlner, ICBM, Oldenburg, Germany

This is a provisional file, not the final typeset article

1.2 Chromatographic programs used in analysis of *Paracoccus* culture extracts

Supplement T 2: Solvent gradient used in LC/MS for analysis of crude extracts from *Paracoccus* spp. (FA) formic acid

Time [min]	Solvent [%]		2% FA in H_2O
	H_2O	CH_3CN	
1.5	90	5	5
18	55	40	5
21	0	95	5
26	0	95	5
27	0	100	0
31	0	100	0
32	90	5	5
35	90	5	5

Supplement T 3: Solvent gradient used in HPLC for fractionation of *Paracoccus marcusii* CP157 crude extracts. The solvent flow rate was 4.75 ml/min.

Time [min]	Solvent [%]	
	H_2O	CH_3CN
0	95	5
1.5	95	5
12	5	95
14	5	95
15	0	100
18	0	100
20	95	5
25	95	5

1.3 *In silico* DNA-DNA hybridization between the genomes of CP157 and closely related reference strains

Supplement T 4: Results of *in silico* DNA-DNA-hybridization based on formula 2 of the GGDC (http://ggdc.dsmz.de/ggdc.php#). Genomes are available on NCBI under the accession numbers PRJNA693451 (*P. marcusii*) GCA_006239215.1 (*P. haeundaensis*), GCA_005048265.1 (*P. hibiscisoli*), GCA_004010775.1 (Arc-7-R13) and CP065892-CP065914 (CP157) Horizontal: reference genomes, vertical: query genomes

	P. marcusii **CP157**	***P. marcusii*** **DSM11574T**	***P. haeundaensis*** **CGMCC 1.8012**	***P. hibiscisoli*** **KACC 18933T**	***Paracoccus*** **sp. Arc7-R13**
P. marcusii **CP157**	-	76.6	77.9	31.2	77
P. marcusii **DSM11574T**	76.6	-	76.9	31.1	78.9
P. haeundaensis **CGMCC 1.8012**	77.9	76.9	-	31.1	77.6
P. hibiscisoli **KACC 18933T**	31.2	31.1	31.1	-	31.3
Paracoccus **sp. Arc7-R13**	77	78.9	77.6	31.3	-

1.4 General properties, genomic Islands (GIs) and prophage regions of the CP157 genome

Supplement T 5: General properties of the complete genome of *Paracoccus marcusii* CP157 consisting of one chromosome (c) and 22 plasmids (p). All sequences are deposited at the NCBI.

Element	Size [bp]	G+C content [%]	Signal peptides	Total genes	CDS	Accession number
**cCP157**	3065500	67.6	296	3073	2981	CP065892
**pCP157_01**	254393	68.5	25	228	228	CP065893
**pCP157_02**	225058	67.7	17	201	200	CP065894
**pCP157_03**	158154	58.7	7	154	154	CP065895
**pCP157_04**	96460	57.7	11	92	92	CP065896
**pCP157_05**	94950	59.6	8	88	88	CP065897
**pCP157_06**	41741	56.4	2	33	33	CP065898
**pCP157_07**	30021	60.3	1	27	27	CP065899
**pCP157_08**	15269	53.2	0	17	17	CP065900
**pCP157_09**	11943	60.5	1	13	13	CP065901
**pCP157_10**	11211	57.5	1	14	14	CP065902
**pCP157_11**	10501	53.2	0	11	11	CP065903
**pCP157_12**	8745	60.8	0	8	8	CP065904
**pCP157_13**	8491	51.9	0	9	9	CP065905
**pCP157_14**	8099	54	1	12	12	CP065906
**pCP157_15**	6778	57	0	8	8	CP065907
**pCP157_16**	6378	59.1	0	4	4	CP065908
**pCP157_17**	6050	58	0	5	5	CP065909
**pCP157_18**	4977	62.4	0	5	5	CP065910
**pCP157_19**	4405	58.2	0	6	6	CP065911
**pCP157_20**	3786	57.7	0	4	4	CP065912
**pCP157_21**	3157	59.6	0	3	3	CP065913
**pCP157_22**	1211	64.3	0	1	1	CP065914

Supplement T 6: Genomic islands (GIs) identified in the genome of CP157 by at least one algorithm applied by IslandViewer 4. Regions overlapping with prophage regions are indicated in *comments*.

GI	Position (from)	Position (to)	Length [kbp]	Detection Method	Comments
1	CP157_00059	CP157_00061	4.5	SIGI-HMM	
2	CP157_01100	CP157_01201	80.7	IslandPath-DIMOB (+SIGI-HMM)	includes Prophage 1
3	CP157_01239	CP157_01260	20.0	IslandPath-DIMOB (+IslandPick)	includes Prophage 2
4	CP157_02008	CP157_02025	16.9	IslandPath-DIMOB (+SIGI-HMM)	
5	CP157_02148	CP157_02170	21.3	IslandPath-DIMOB (+IslandPick+SIGI-HMM)	
6	CP157_02197	CP157_02218	27.7	IslandPath-DIMOB	
7	CP157_02232	CP157_02256	18.1	IslandPath-DIMOB (+SIGI-HMM)	includes Prophage 4
8	CP157_02649	CP157_02661	9.1	IslandPath-DIMOB	

This is a provisional file, not the final typeset article

Supplement T 7: Prophage elements detected in the genome of CP157 by PHASTER and their location on the chromosome (c) or a plasmid (p). Prophages with scores >90 are considered as intact by PHASTER. Hit proteins: Number of proteins in the region with matches in the phage protein database. Shared proteins: Highest number of proteins shared between CP157 genome and a phage in the database.

Prophage	Location	Position (from)	Position (to)	Length [kbp]	Completeness [score]	Hit proteins	Shared proteins
1	cCP157	CP157_01129	CP157_01149	16.4	incomplete (40)	12	4
2	cCP157	CP157_01241	CP157_01247	5.4	incomplete (60)	6	5
3	cCP157	CP157_01816	CP157_01834	15.1	incomplete (40)	14	5
4	cCP157	CP157_02238	CP157_02246	6.9	incomplete (50)	8	3
5	pCP157_03	CP157_03561	CP157_03572	16.4	questionable (80)	13	3
6	pCP157_05	CP157_03796	CP157_03807	11.3	questionable (70)	9	3
7	pCP157_05	CP157_03853	CP157_03859	4.5	incomplete (60)	6	3
8	pCP157_06	CP157_03893	CP157_03900	6	incomplete (30)	6	1

1.5 AntiSMASH database entries for diverse *Paracoccus* spp. and comparison with CP157

Supplement T 8: Overview of BSGCs found in antiSMASH database entries for the genus *Paracoccus*. Results obtained for manual search against the CP157 genome are indicated (°). Database entries marked with * were added before the latest update of the database (September 2020). All other entries were obtained from the antiSMASH database 3 (January 2021). Hybrid clusters mostly harbor features of NRPS-PKS-HSR. Type strains are marked with T. Abbreviations: (HSR) homoserine lactone, (T1PKS) type 1 polyketide synthase, (T3PKS) type 3 polyketide synthase, (TP) terpene, (E) ectoine, (SDP) siderophore, (RiPP) ribosomally synthesized and post-translationally modified peptide, (LP) lasso peptide, (BL) butyrolactone, (ThP) thiopeptide, (BL) betalactone

Strain	HSR	T1PKS	T3PKS	TP	E	SDP	NRPS	Bacteriocin	Hybrid	LP	BL	ThP	BL	RiPP	Other
***Paracoccus marcusii* CP157**	**1**		**1**	**1**	**1**	**1**		**1**	**1**				**1**		
Paracoccus alcaliphilus DSM 8512T*	1	1			1		1		1						1
Paracoccus alkenifer DSM 11593T*	1	1					1								
Paracoccus aminophilus JCM 7686T	2						2	1	5		1	1			
Paracoccus aminovorans JCM7685T	1						3	2	1						
Paracoccus chinensis CGMCC 1.7655T*	1		1	1			1			1					
Paracoccus contaminans RKI 16-01929T	1									1			1		
Paracoccus denitrificans PD1222	1	1					3	1	2					1	
Paracoccus halophilus CGMCC 1.6117T	2				1	1	2		1						
Paracoccus haeundaensis CCGMCC 1.8012°	2		1	1	1	1		1	1				1		
Paracoccus hibiscisoli CCTCC AB2016182T°	2	1		1	1	1		1							
Paracoccus homiensis DSM 17862T*	1				1			1							
Paracoccus isoporae DSM 22220T*	1				1										
Paracoccus jeotgali CBA4604T	1		1		1				1						
Paracoccus kondratievae BJQ0001	1						3		1					1	
Paracoccus liaowanqingii 2251T	2				1		1	1	2						
Paracoccus limosus JCM 17370T	1						3	2	1					1	
Paracoccus lutimaris CECT 8525T	1						1	2	2				1		
Paracoccus marcusii DSM 11574T°	2		1	1	1	1		1					1		
Paracoccus mutanolyticus RSP-02T	1								1						
Paracoccus pantotrophus DSM 2944T	1						4							1	
Paracoccus ravus YJ057T	1						2	1	1						
Paracoccus saliphilus DSM 18447T	1				1	1	1	1					2		
Paracoccus sanguinis OM2	1		1						1				1		

Strain	HSR	T1PKS	T3PKS	TP	E	SDP	NRPS	Bacteriocin	Hybrid	LP	BL	ThP	BL	RiPP	Other
Paracoccus solventivorans DSM 6637T*	1	1													
Paracoccus sp. 228	3		1	1	1	1		1	1				1	1	
Paracoccus sp. AK26	2			1											
Paracoccus sp. Arc7-R13°	3			1	1	1		1	1				1		
Paracoccus sp. BM15	3	1	1		1			1	2				1		
Paracoccus sp. CBA4604*	1		1		1				1						
Paracoccus sp. DK608	1						3		1						
Paracoccus sp. J39	1	1					3	1	1						
Paracoccus sp. J55	1	1					3		1						
Paracoccus sp. J56	1						3	1	1					1	
Paracoccus sp. JM45	3		1		1	1	1	1					1		
Paracoccus sp. MKU1*	1	1					1		1						
Paracoccus sp. N5*	1	1					1	1							
Paracoccus sp. PAMC 22219*	1	1	1	1	1	1	1	1							
Paracoccus sp. S1 E-3	1			1			1	1							
Paracoccus sp. S4493*	1			1			1	1	1						1
Paracoccus sp. SC-6	1		1										1		
Paracoccus sp. SM22M-07	4				1	1		1		1			1		
Paracoccus sp. TRP*	1	1					1								
Paracoccus sphaerophysae HAMBI 3106T*	1			1											
Paracoccus sulfuroxidans CGMCC 1.5364T	1						2	1	2						
Paracoccus thiocyanatus ATCC 700171T	1						2		1					1	
Paracoccus tibetensis CGMCC 1.8925T	1			1	1				1				2		
Paracoccus versutus DSM 582T*	1	1					1	1							
Paracoccus yeei ATCC BAA-599T°	1	1		1			3				1				1
Paracoccus zeaxanthinifaciens ATCC 21588T	1	1		1	1	1		1							
Paracoccus zhejiangensis J6T	3						2	2	1		1				

1.6 AntiSMASH comparison of CP157 and closely related *Paracoccus* strains

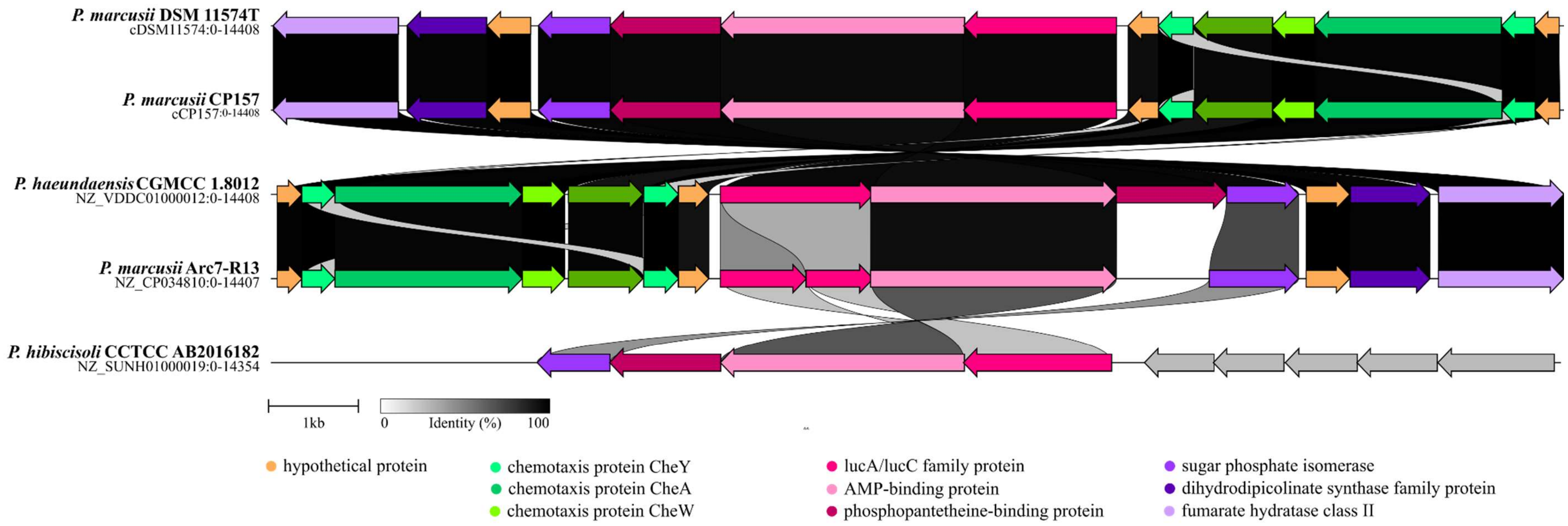

Supplement F 1: Comparison of the siderophore clusters found in the strains CGMCC 1.8012, CP157 and Arc7-R13 and the type strains of *Paracoccus marcusii* and *P. hibiscisoli* by antiSMASH 5.2. While highly similar synteny is shared between the clusters of the first four strains (DDH of genomes >70 %), the cluster found in *P. hibiscisoli* (DDH=31.1 %) shows marked differences in organization and gene content. The comparison of siderophore clusters visualized here is given as an example. Other BSGCs found in all five strains (bacteriocin, homoserine lactone, hybrid clusters) relate to each other in the same manner. Query genomes for CGMCC 1.8012, Arc7-R13 and *P. hibiscisoli* were accessed from NCBI. The figure was generated using clinker according to Gilchrist and Chooi (2021).

This is a provisional file, not the final typeset article

1.7 Antibacterial activity of *Paracoccus* spp. crude extracts

Supplement T 9: Antimicrobial activity of *Paracoccus* spp. culture extracts (XAD-7/XAD-16). Strains tested for susceptibility to the extracts originate from the carapace of *C. pagurus* (CP) or were obtained from the DSMZ. Inhibition zones are referred to as radius in [mm] and are based on mean values (n=3). Extracted MB medium and methanol were tested as negative controls and did not show any impact on target strain growth. (-) no inhibition observed; inhibition zone: (+) <1 mm, (++) 1-3 mm, (+++) >3 mm. T type strain.

			**Target**				
			**Alphaproteobacteria**			**Flavobacteriia**	
	**ID**	**Closest relative**	***Paracoccus sp.* CP32**	***Paracoccus sp.* CP35**	***Paracoccus sp.* CP157**	***Tenacibaculum sp.* CP14**	***Aquimarina sp.* CP51**
**Producer**	3501	*P. marcusii*	++/++	++/++			++/-
	49B05	*P. liaowanqingii*	++/++	++/++			++/+
	C13	*P. aquimaris*	+++/++	++/++	++/++		++/++
	CP137	*P. yeei*	++/++	++/++			-/-
	CP157	*P. marcusii*	+++/+++	++/++	-/-	++/++	++/-
	CP32	*P. marcusii*	+++/++	+++/++	-/-	++/++	++/-
	CP35	*P. marcusii*	+++/++		-/-	++/++	+/-
	GWS-BW-H72M	*P. marcusii*	++/++	++/++			-/-
	GWS-SE-H131	*P. hibiscisoli*	++/+	++/++			-/++
	**ID**	**Type strain**					
**Producer**	DSM 19484T	*P. aestuarii*	+/+	+/+			-/-
	DSM 8512T	*P. alcaliphilus*	++/++	++/++	++/++		+/+
	KCTC 22803T	*P. fistulariae*	++/++				++/-
	LMG P-21903T	*P. haeundaensis*	+++/+++	+++/++	+++/++		+++/++
	KACC 18933T	*P. hibiscisoli*	+++/++	+++/++			-/-
	DSM 11574T	*P. marcusii*	+++/++	+++/++	-/-		++/++
	DSM 18447T	*P. saliphilus*	-/-	-/-			-/-
	DSM 14827T	*P. seriniphilus*	+++/+++	+++/++	+++/+++		++/+++
	LMG 25392T	*P. stylophorae*	++/++	++/++	++/++		++/++

			**Bacilli**		**Gammaproteobacteria**		**Actinobacteria**
	**ID**	**Closest relative**	***Bacillus subtilis* SMS4**	***Escherichia coli* DSM 613**	***Pseudomonas stutzeri* DSM 5190**	***Raoultella planticola* DSM 3069**	***Arthrobacter sp.* CP30**
**Producer**	3501	*P. marcusii*					++/-
	49B05	*P. marcusii*					++/+
	C13	*P. aquimaris*					++/++
	CP137	*P. yeei*					-/-
	CP157	*P. marcusii*	++/++	-/-	++/++	++/+	++/++
	CP32	*P. carotinifaciens*					+++/++
	CP35	*P. marcusii*					-/-
	GWS-BW-H72M	*P. marcusii*					-/-
	GWS-SE-H131	*P. hibiscisoli*					-/++
	**ID**	**Type strain**					
**Producer**	DSM 19484T	*P. aestuarii*					-/-
	DSM 8512T	*P. alcaliphilus*					-/-
	KCTC 22803T	*P. fistulariae*					-/-
	LMG P-21903T	*P. haeundaensis*					+++/++
	KACC 18933T	*P. hibiscisoli*					+/-
	DSM 11574T	*P. marcusii*					++/++
	DSM 18447T	*P. saliphilus*					-/-
	DSM 14827T	*P. seriniphilus*					+++/++
	LMG 25392T	*P. stylophorae*					-/-

1.8 Antialgal activity of CP157

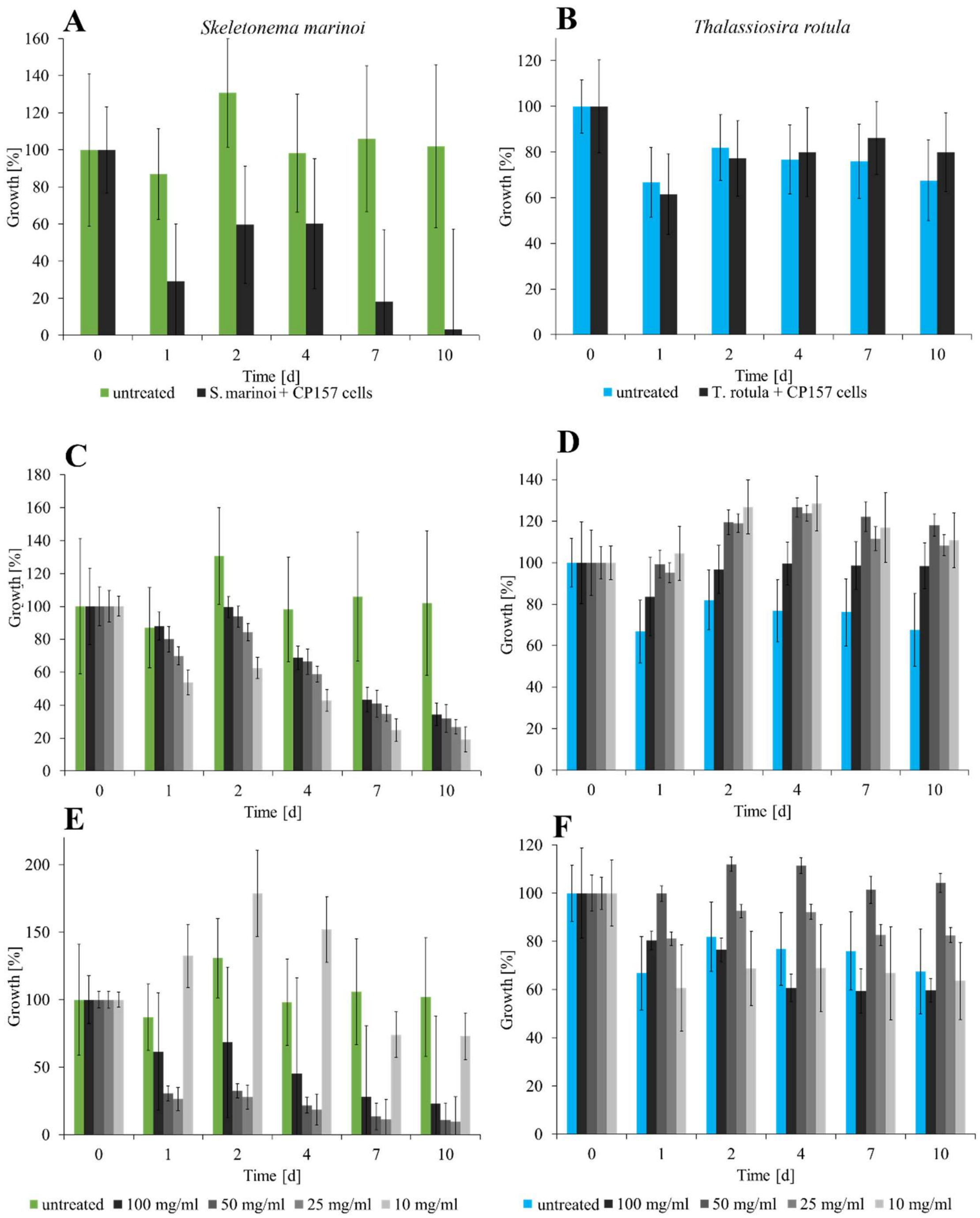

Supplement F 2: Impact of CP157 crude extracts (E, F) and living culture (A, B) respectively on growth of microalgae *Thalassiosira rotula* (blue) and *Skeletonema marinoi* (green) and effect of extracted culture medium (MB; C, D) on microalgae. Growth was determined by measuring relative fluorescence over time. Changes are expressed as percentage relative to the fluorescence measured at t_0. Values are means ± standard deviation (n=3).

This is a provisional file, not the final typeset article

1.9 Antilarval activity of CP157

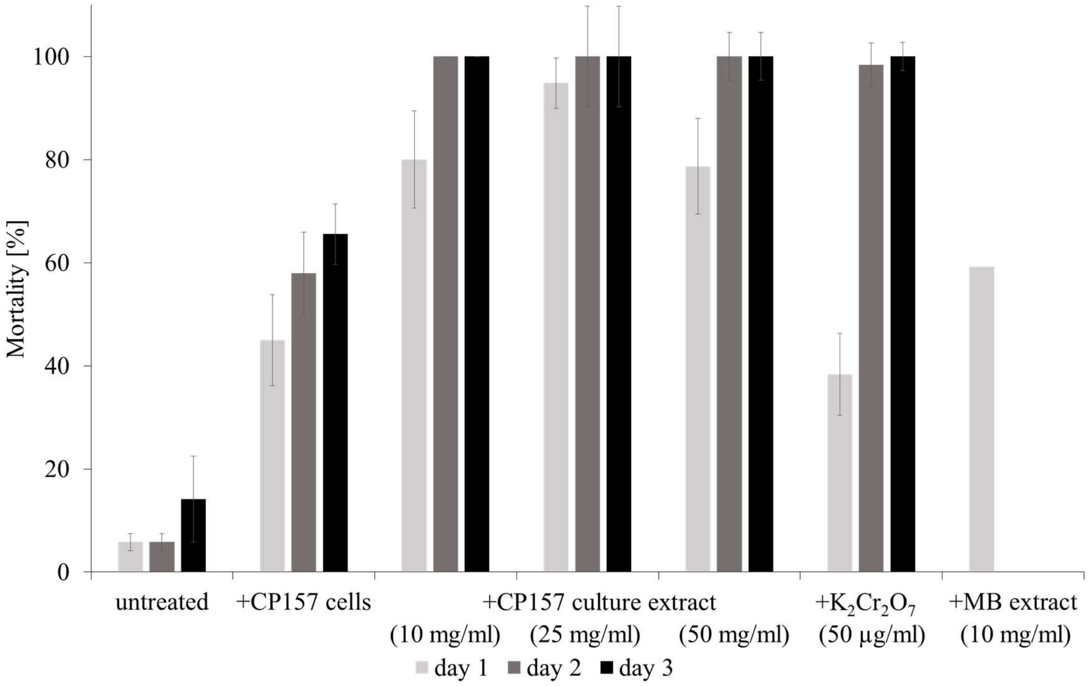

Supplement F 3: Mortality of untreated *A. salina* larvae and after treatment with *Paracoccus* sp. CP157 culture extract or cells or extracted medium. Impact of the lowest active concentration of MB extract is shown exemplarily. Potassium dichromate served as a positive control for larvicidal activity. Values are means ± standard deviation (n=3).

1.10 Self-resistance to antimicrobials produced by *Paracoccus* spp. as common but not ubiquitous trait

Target strain	Producer strain: CP157	*P. haeundaensis*	*P. marcusii*	*P. alcaliphilus*	*P. seriniphilus*	*P. stylophorae*
**CP157**	-	+++	-	++	+++	++
P. haeundaensis	++	+++	+++		+++	++
P. marcusii	++	+++	+++		++	++
P. alcaliphilus	-	++	++	++	-	-
P. seriniphilus	-	-	-	+++	-	-
P. stylophorae	-	++	+++	++	-	-

Target strain	Producer strain: CP157	*P. haeundaensis*	*P. marcusii*	*P. alcaliphilus*	*P. seriniphilus*	*P. stylophorae*
**CP157**	-	++	-	++	+++	++
P. haeundaensis	++	++	++		+++	++
P. marcusii	++	++	++		++	++
P. alcaliphilus	-	++	++	++	-	-
P. seriniphilus	-	-	-	-	-	-
P. stylophorae	-	+++	++	++	-	-

Supplement F 4: Results of cross-tests between culture extracts from *P. marcusii* CP157 and different *Paracoccus* type strains prepared with XAD-7 (left) and XAD-16 (right). Size of inhibition zones < 1mm (+), 1-3 mm (++), >3 mm (+++), no inhibition (-), hatched fields: combination not tested.

This is a provisional file, not the final typeset article

1.11 LC/MS analysis of culture extracts from 18 *Paracoccus* spp.

Supplement T 10: Number of compounds detected in *Paracoccus* culture extracts via LC/MS. Masses found in extracted medium control and signals with intensities <10 000 were removed as medium components or background noise, respectively. The total number of different peaks identified in extracts prepared with XAD-7 or -16 is given in the last row. Strains are arranged from top to bottom with decreasing 16S rRNA gene sequence similarity to *Paracoccus marcusii* CP157. T type strains

Organism		Detected Compounds [#]	
**Strain**	**Species**	**XAD-7**	**XAD-16**
**CP157**	***P. marcusii***	**469**	**535**
CP32	*Paracoccus* sp.	548	550
CP35	*Paracoccus* sp.	574	557
DSM 11574T	*P. marcusii*	577	669
GWS-BW-H72M	*Paracoccus* sp.	898	720
3501	*Paracoccus* sp.	525	771
LMG P-21903T	*P. haeundaensis*	612	682
GWS-SE-H131	*Paracoccus* sp.	525	705
KACC 18933T	*P. hibiscisoli*	526	737
49B05	*Paracoccus* sp.	625	639
DSM 14827T	*P. seriniphilus*	497	893
DSM 19484T	*P. aestuarii*	500	665
CP137	*Paracoccus* sp.	381	1013
DSM 8512T	*P. alcaliphilus*	490	648
C13	*Paracoccus* sp.	786	748
DSM 18447T	*P. saliphilus*	754	837
LMG 25392T	*P. stylophorae*	582	767
KCTC 22803T	*P. fistulariae*	865	966
**Total**	**Different peaks**	2589	2386

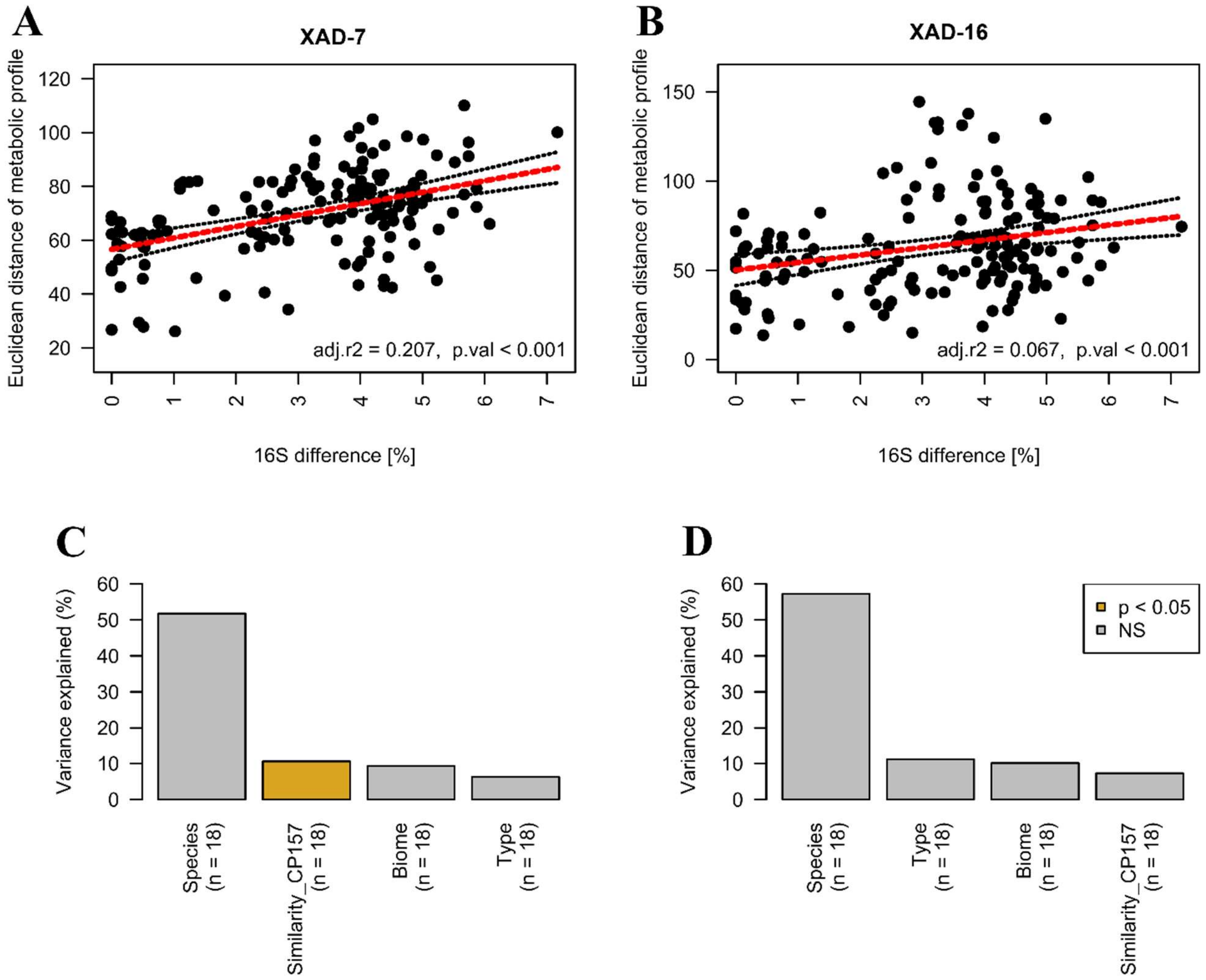

Supplement F 5: PERMANOVA results showing a weak correlation between metabolic profile of *Paracoccus* XAD-7 extracts and the phylogenetic distance of the respective producer strains (n=18; A). No correlation with phylogenetic distance was found with XAD-16 extracts (B). No significant correlation was found between metabolic profile and habitat ("biome") or lifestyle (attached/free-living; "type") was found in XAD-7 (C) or XAD-16 (D) extracts.

This is a provisional file, not the final typeset article

1.12 Bioactive fractions prepared via HPLC from CP157 crude extracts

Supplement T 11: Fractions of CP157 culture extracts (XAD-7, MB) prepared via HPLC and their antibacterial activity against three different target strains. The degree of activity is given as mean (n=3): (-) no inhibition observed; inhibition zone: (+) <1 mm, (++) 1-3 mm, (+++) >3 mm; (n.d.) not determined

**Fraction**	**Target**		
	***Paracoccus* sp.**		***Arthrobacter* sp.**
	**CP35**	**CP32**	**CP30**
F3	-	n.d.	n.d.
F4	++	+	++
F5	++	++	++
F6	++	+	+
F7	-	-	-
F8	++	+	+
F9	++	+	++
F10	++	++	+++
F11	+++	n.d.	-
F12	-	n.d.	-
F13	++	-	-
F14	-	-	-
F15	++	-	-

2 References

Bagley, S.T.,Seidler, R.J. and Brenner, D.J. (1981): Klebsiella planticola sp. nov.: A new species of enterobacteriaceae found primarily in nonclinical environments. *Curr Microbiol* 6 (2), 105–109. DOI: 10.1007/BF01569013.

Burri, R. and Stutzer, A. (1895): Ueber Nitrat zerstörende Bakterien und den durch dieselben bedingten Stickstoffverlust. *Zentralbl. Bakteriol. Parasitenkd. Abt. II* 1, 1895, 257–265.

Daegelen, P.,Studier, F.W.,Lenski, R.E.,Cure, S. and Kim, J.F. (2009): Tracing ancestors and relatives of Escherichia coli B, and the derivation of B strains REL606 and BL21(DE3). *Journal of Molecular Biology* 394 (4), 634–643. DOI: 10.1016/j.jmb.2009.09.022.

Dogs, M.,Wemheuer, B.,Wolter, L.,Bergen, N.,Daniel, R.,Simon, M. and Brinkhoff, T. (2017): Rhodobacteraceae on the marine brown alga Fucus spiralis are abundant and show physiological adaptation to an epiphytic lifestyle. *Systematic and applied microbiology* 40 (6), 370–382. DOI: 10.1016/j.syapm.2017.05.006.

Fu, Y.,Rivkin, R.B. and Lang, A.S. (2019): Effects of Vertical Water Mass Segregation on Bacterial Community Structure in the Beaufort Sea. *Microorganisms* 7 (10). DOI: 10.3390/microorganisms7100385.

Gilchrist, C.L.M. and Chooi, Y.-H. (2021): Clinker & clustermap.js: Automatic generation of gene cluster comparison figures. *Bioinformatics (Oxford, England)*. DOI: 10.1093/bioinformatics/btab007.

Harker, M.,Hirschberg, J. and Oren, A. (1998): Paracoccus marcusii sp. nov., an orange gram-negative coccus. *International journal of systematic bacteriology* 48 Pt 2, 543–548. DOI: 10.1099/00207713-48-2-543.

Kim, Y.-O.,Kong, H.J.,Park, S.,Kang, S.-J.,Kim, K.-K.,Moon, D.Y. *et al.* (2010): Paracoccus fistulariae sp. nov., a lipolytic bacterium isolated from bluespotted cornetfish, Fistularia commersonii. *International journal of systematic and evolutionary microbiology* 60 (Pt 12), 2908–2912. DOI: 10.1099/ijs.0.021808-0.

Lee, J.H.,Kim, Y.S.,Choi, T.-J.,Lee, W.J. and Kim, Y.T. (2004): Paracoccus haeundaensis sp. nov., a Gram-negative, halophilic, astaxanthin-producing bacterium. *International journal of systematic and evolutionary microbiology* 54 (Pt 5), 1699–1702. DOI: 10.1099/ijs.0.63146-0.

Lin, P.,Yan, Z.-F.,Won, K.-H.,Yang, J.-E.,Li, C.-T.,Kook, M. *et al.* (2017): Paracoccus hibiscisoli sp. nov., isolated from the rhizosphere of Mugunghwa (Hibiscus syriacus). *International journal of systematic and evolutionary microbiology* 67 (7), 2452–2458. DOI: 10.1099/ijsem.0.001990.

Pukall, R.,Laroche, M.,Kroppenstedt, R.M.,Schumann, P.,Stackebrandt, E. and Ulber, R. (2003): Paracoccus seriniphilus sp. nov., an L-serine-dehydratase-producing coccus isolated from the marine bryozoan Bugula plumosa. *International journal of systematic and evolutionary microbiology* 53 (Pt 2), 443–447. DOI: 10.1099/ijs.0.02352-0.

Roh, S.W.,Nam, Y.-D.,Chang, H.-W.,Kim, K.-H.,Kim, M.-S.,Shin, K.-S. *et al.* (2009): Paracoccus aestuarii sp. nov., isolated from tidal flat sediment. *International journal of systematic and evolutionary microbiology* 59 (Pt 4), 790–794. DOI: 10.1099/ijs.0.65759-0.

Sheu, S.-Y.,Jiang, S.-R.,Chen, C.A.,Wang, J.-T. and Chen, W.-M. (2011): Paracoccus stylophorae sp. nov., isolated from the reef-building coral Stylophora pistillata. *International journal of systematic and evolutionary microbiology* 61 (Pt 9), 2221–2226. DOI: 10.1099/ijs.0.028035-0.

Stevens, H.,Stübner, M.,Simon, M. and Brinkhoff, T. (2005): Phylogeny of Proteobacteria and Bacteroidetes from oxic habitats of a tidal flat ecosystem. *FEMS Microbiology Ecology* 54 (3), 351–365. DOI: 10.1016/j.femsec.2005.04.008.

URAKAMI, T.,TAMAOKA, J.,Suzuki, K.I. and KOMAGATA, K. (1989): Paracoccus alcaliphilus sp. nov., an Alkaliphilic and Facultatively Methylotrophic Bacterium. *International journal of systematic bacteriology* 39 (2), 116–121. DOI: 10.1099/00207713-39-2-116.

Wang, Y.,Tang, S.-K.,Lou, K.,Mao, P.-H.,Jin, X.,Jiang, C.-L. *et al.* (2009): Paracoccus saliphilus sp. nov., a halophilic bacterium isolated from a saline soil. *International journal of systematic and evolutionary microbiology* 59 (Pt 8), 1924–1928. DOI: 10.1099/ijs.0.005918-0.
