# Supplementary material for: High Potential for Secondary Metabolite Production of Paracoccus marcusii CP157, Isolated From the Crustacean Cancer pagurus
Source: Front Microbiol. 2021 Jun 28;12:688754. doi: 10.3389/fmicb.2021.688754 (PMC8273931; doi:10.3389/fmicb.2021.688754)
Supplement: Supplementary file 2 [file Data_Sheet_2.pdf]

1 Locus Tags

Supplement 2 1: List of key genes within BSGCs detected by antiSMASH 5.2 in the genome of CP157. Direct pBLAST was conducted using CP157 genes as query against reference genes from the MIBiG database or UniProt respectively.

Genome		AntiSMASH	pBLAST				Direct pBLAST		
Locus Tag	Annotation	Cluster type	Description	Best hit	Similarity [%]	Accession	Reference gene	Similarity [%]	Coverage [%]
CP157_00998	3-dehydroshikimate dehydratase	siderophore	sugar phosphate isomerase/epimerase	*Paracoccus haeundaensis*	98	WP_045998840.1	asbF (MIBiG)	33	96
CP157_00999	hypothetical	siderophore	phosphopantetheine-binding protein	*Paracoccus haeundaensis*	98	WP_139598365.1	asbE (MIBiG)	33	96
CP157_01000	N(2)-citryl-N(6)-acetyl-N(6)-hydroxylysine synthase	siderophore	AMP-binding protein	*Paracoccus* sp. 228	98	WP_045998841.1	asbB (MIBiG)	27	93
CP157_01001	N(2)-citryl-N(6)-acetyl-N(6)-hydroxylysine synthase	siderophore	IucA/IucC family siderophore biosynthesis protein	*Paracoccus* sp. 228	97	WP_052715057.1	asbA (MIBiG)	27	98
CP157_01019	L-ectoine synthase	ectoine	ectoine synthase	*Paracoccus* sp. 228	99	WP_045998850.1	ectC (MIBiG)	91	96
CP157_01020	Diaminobutyrate--2-oxoglutarate transaminase	ectoine	diaminobutyrate--2-oxoglutarate transaminase	*Paracoccus* sp. 228	99	WP_045998851.1	ectB (MIBiG)	81	100
CP157_01021	L-2,4-diaminobutyric acid acetyltransferase	ectoine	diaminobutyrate acetyltransferase	*Paracoccus marcusii*	99	MQN29534.1	ectA (MIBiG)	75	99
CP157_01022	Transcriptional regulator MprA	ectoine	MarR family transcriptional regulator	*Paracoccus marcusii*	99	WP_139084946.1	ectR (MIBiG)	66	88
CP157_01581	Protein YhgF	betalactone							
CP157_01582	Sulfoquinovose isomerase	betalactone							
CP157_01583	putative sensor-histidine kinase	betalactone	sensor histidine kinase	*Paracoccus marcusii*	90	MQN29355.1			

Genome		AntiSMASH	pBLAST				Direct pBLAST		
**Locus Tag**	**Annotation**	**Cluster type**	**Description**	**Best hit**	**Similarity [%]**	**Accession**	**Reference gene**	**Similarity [%]**	**Coverage [%]**
CP157_01584	hypothetical	betalactone							
CP157_01585	cobalamin biosynthesis protein	betalactone							
CP157_01586	hypothetical	betalactone							
CP157_01587	Acyl-CoA dehydrogenase	betalactone	isovaleryl-CoA dehydrogenase	*Paracoccus haeundaensis*	99	WP_139598613.1			
CP157_01588	Acetyl-coenzyme A synthetase	betalactone	AMP-binding protein	*Paracoccus haeundaensis*	98	WP_139598612.1			
CP157_01589	Methylmalonyl-CoA carboxyltransferase 12S subunit	betalactone	methylcrotonoyl-CoA carboxylase	*Paracoccus* sp. 228	100	WP_045999150.1			
CP157_01590	Acetyl-/propionyl-coenzyme A carboxylase alpha chain	betalactone	biotin/lipoyl-binding protein	*Paracoccus haeundaensis*	97	WP_139598610.1			
CP157_01591	3-hydroxy-3-isohexenylglutaryl-CoA/hydroxy-methylglutaryl-CoA lyase	betalactone	hydroxymethylglutaryl-CoA lyase	*Paracoccus haeundaensis*	96	WP_139598609.1			
CP157_01592	putative enoyl-CoA hydratase echA8	betalactone	crotonase/enoyl-CoA hydratase family protein	*Paracoccus* sp. S4493	98	WP_045983026.1			
CP157_01593	Divalent metal cation transporter MntH	betalactone	divalent metal cation transporter	*Paracoccus* sp. PAMC22219	95	WP_156117970.1			
CP157_01594	Serine hydroxymethyltransferase 1	betalactone	aminotransferase class I/II-fold pyridoxal phosphate-dependent enzyme	*Paracoccus marcusii*	100	WP_139085068.1			
CP157_01595	NAD kinase	betalactone							
CP157_01596	hypothetical	betalactone							

This is a provisional file, not the final typeset article

Genome		AntiSMASH	pBLAST				Direct pBLAST		
**Locus Tag**	**Annotation**	**Cluster type**	**Description**	**Best hit**	**Similarity [%]**	**Accession**	**Reference gene**	**Similarity [%]**	**Coverage [%]**
CP157_01597	RNA polymerase sigma-32 factor	betalactone							
CP157_01598	Ribosomal large subunit pseudouridine synthase D	betalactone							
CP157_01599	hypothetical	betalactone							
CP157_01600	hypothetical	betalactone							
CP157_01601	Prolipoprotein diacylglyceryl transferase	betalactone							
CP157_02139	Tryptophan--tRNA ligase	carotenoid (terpene)							
CP157_02140	C4-dicarboxylic acid transporter DauA	carotenoid (terpene)							
CP157_02141	hypothetical	carotenoid (terpene)							
CP157_02142	hypothetical	carotenoid (terpene)							
CP157_02143	hypothetical	carotenoid (terpene)							
CP157_02144	hypothetical	carotenoid (terpene)							
CP157_02145	D-inositol-3-phosphate glycosyltransferase, mshA_3	carotenoid (terpene)	glycosyltransferase	*Paracoccus haeundaensis*	99.5	WP_139597671.1			
CP157_02146	Geranylgeranyl diphosphate synthase, crtE	carotenoid (terpene)	MULTISPECIES: polyprenyl synthetase family protein	Proteobacteria	99	WP_052714724.1	crtE (MIBiG)	99	100
CP157_02147	15-cis-phytoene synthase	carotenoid (terpene)	squalene/phytoene synthase family protein	*Paracoccus haeundaensis*	99	WP_139597669.1	crtB (UniProt)	98	99

Genome		AntiSMASH	pBLAST				Direct pBLAST		
**Locus Tag**	**Annotation**	**Cluster type**	**Description**	**Best hit**	**Similarity [%]**	**Accession**	**Reference gene**	**Similarity [%]**	**Coverage [%]**
CP157_02148	Phytoene desaturase (lycopene-forming)	carotenoid (terpene)	phytoene desaturase	*Paracoccus marcusii*	100	WP_139085606.1	crtI (UniProt)	97	100
CP157_02149	hypothetical	carotenoid (terpene)	lycopene beta-cyclase CrtY	*Paracoccus haeundaensis*	98	WP_139597667.1	crtY (UniProt)	98	100
CP157_02150	hypothetical	carotenoid (terpene)	carotene hydroxylase	*Paracoccus marcusii*	100	WP_127898118.1	crtZ (MIBiG)	100	100
CP157_02151	hypothetical	carotenoid (terpene)	beta-carotene ketolase	*Paracoccus* sp. SS4493	99	WP_045982217.1	crtW (MIBiG)	97	100
CP157_02177	hypothetical	T3PKS							
CP157_02178	hypothetical	T3PKS							
CP157_02179	Cellulose synthase 1	T3PKS	glycosyltransferase	*Paracoccus* sp. S4493	99	WP_045983202.1			
CP157_02180	hypothetical	T3PKS	acyltransferase	*Paracoccus* sp. S4493	99.7	WP_045983208.1			
CP157_02181	hypothetical	T3PKS							
CP157_02182	Anti-anti-sigma-B factor	T3PKS							
CP157_02183	Transcriptional regulatory protein LiaR	T3PKS	LuxR family two component transcriptional regulator	*Pseudomonas stutzeri*	99	TYP68849.1			
CP157_02184	hypothetical	T3PKS							
CP157_02185	Autoinducer 2 sensor kinase/phosphatase LuxQ	T3PKS	response regulator	*Paracoccus* sp. 228	99	WP_052715284.1			
CP157_02186	hypothetical	T3PKS							
CP157_02187	Bicarbonate transport ATP-binding protein CmpC	T3PKS	ABC transporter ATP-binding protein	*Paracoccus* sp. PAMC 22219	90	WP_042244963.1			
CP157_02188	hypothetical	T3PKS	nuclear transport factor 2 family protein	*Paracoccus haeundaensis*	99	WP_139597647.1			

This is a provisional file, not the final typeset article

Genome		AntiSMASH	pBLAST				Direct pBLAST		
Locus Tag	Annotation	Cluster type	Description	Best hit	Similarity [%]	Accession	Reference gene	Similarity [%]	Coverage [%]
CP157_02189	cyanate hydratase	T3PKS							
CP157_02190	hypothetical	T3PKS							
CP157_02191	Sensor protein QseC	T3PKS	two-component sensor histidine kinase	*Paracoccus* sp. PAMC 22219	88	WP_042244957.1			
CP157_02192	Transcriptional regulatory protein QseB	T3PKS	response regulator transcription factor	*Paracoccus* sp. S4493	99	WP_045984055.1			
CP157_02193	hypothetical	T3PKS	MULTIPSPECIES: thermostable hemolysin	Proteobacteria	98	WP_052714966.1			
CP157_02194	2-succinylbenzoate--CoA ligase	T3PKS	AMP-binding protein	*Paracoccus* sp. S4493	96	WP_045984054.1			
CP157_02195	hypothetical	T3PKS							
CP157_02196	Aklaviketone reductase DauE	T3PKS	SDR family NAD(P)-dependent oxidoreductase	*Paracoccus* sp. S4493	99	WP_045983967.1			
CP157_02197	hypothetical	T3PKS	TauD/TfdA family dioxygenase	*Paracoccus haeundaensis*	98	WP_139597644.1			
CP157_02198	hypothetical	T3PKS							
CP157_02199	1,3,6,8-tetrahydroxynaphthalene synthase	T3PKS	type III polyketide synthase	*Paracoccus haeundaensis*	99	WP_139597642.1			
CP157_02200	hypothetical	T3PKS	membrane protein	*Paracoccus* sp. S4493	97	WP_045983835.1			
CP157_02201	Zinc/cadmium/lead-transporting P-type ATPase	T3PKS	cadmium-translocating P-type ATPase	*Paracoccus* sp. 228	98	WP_046001141.1			
CP157_02202	HTH-type transcriptional regulator ZntR	T3PKS	MerR family DNA-binding protein	*Paracoccus marcusii*	98	MQN29080.1			
CP157_02203	Sensor histidine kinase RegB	T3PKS	MerR family DNA-binding protein	*Paracoccus marcusii*	98	WP_173403712.1			

Genome		AntiSMASH	pBLAST				Direct pBLAST		
Locus Tag	Annotation	Cluster type	Description	Best hit	Similarity [%]	Accession	Reference gene	Similarity [%]	Coverage [%]
CP157_02204	hypothetical	T3PKS	response regulator	*Paracoccus* sp. S4493	100	WP_045983720.1			
CP157_02205	Photosynthetic apparatus regulatory protein RegA	T3PKS	response regulator	*Paracoccus haeundaensis*	99	WP_139597638.1			
CP157_02206	putative SURF1-like protein	T3PKS	SURF1-like protein	*Paracoccus* sp. 228	99	WP_046000910.1			
CP157_02207	Cytochrome bo(3) ubiquinol oxidase subunit 4	T3PKS							
CP157_02208	Cytochrome bo(3) ubiquinol oxidase subunit 3	T3PKS							
CP157_02209	Cytochrome bo(3) ubiquinol oxidase subunit 1	T3PKS	cytochrome ubiquinol oxidase subunit I	*Paracoccus* sp. S4493	100	WP_045983267.1			
CP157_02210	Cytochrome bo(3) ubiquinol oxidase subunit 2	T3PKS							
CP157_02211	Inner membrane metabolite transport protein YhjE	T3PKS	MFS transporter	*Paracoccus* PAMC 22219	96	WP_042251046.1			
CP157_02212	Putative bifunctional exonuclease/endonuclease protein	T3PKS							
CP157_02213	Leukotoxin	T3PKS	Hemolysin-type calcium-binding repeat-containing protein	*Paracoccus solventivorans*	57	SHL84061.1			
CP157_02214	hypothetical	T3PKS							
CP157_02215	hypothetical	T3PKS							
CP157_02216	hypothetical	T3PKS							

This is a provisional file, not the final typeset article

Genome		AntiSMASH	pBLAST				Direct pBLAST		
Locus Tag	Annotation	Cluster type	Description	Best hit	Similarity [%]	Accession	Reference gene	Similarity [%]	Coverage [%]
CP157_02442	isovaleryl-homoserine lactone synthase	homoserine lactone	N-acyl-L-homoserine lactone synthetase	*Paracoccus haeundaensis*	100	WP_139599344.1			
CP157_02443	hypothetical	homoserine lactone	autoinducer binding domain-containing protein	*Paracoccus* sp. PAMC 22219	99.5	WP_042247919.1			
CP157_03442	hypothetical	hybrid cluster	fumarylacetoacetase	*Paracoccus haeundaensis*	98	WP_139598045.1			
CP157_03443	Delta(1)-pyrroline-2-carboxylate reductase	hybrid cluster	ornithine cyclodeaminase	*Paracoccus* sp. 228	97	WP_045999776.1			
CP157_03444	xylulose kinase	hybrid cluster	xylulokinase	*Paracoccus haeundaensis*	100	WP_139598047.1			
CP157_03445	regulatory protein SdiA	hybrid cluster	LuxR family transcriptional regulator	*Paracoccus* sp. 228	100	WP_052715157.1			
CP157_03446	hypothetical	hybrid cluster	hypothetical						
CP157_03447	Acyl-homoserine-lactone synthase	hybrid cluster	acyl homoserine lactone synthase	*Paracoccus halophilus*	65	SFA58385.1			
CP157_03448	Phosphate-import permease protein PhnE	hybrid cluster	ABC transporter permease	*Paracoccus* sp. S4493	98	WP_045983435.1			
CP157_03453	Formate dehydrogenase-O major subunit precursor	hybrid cluster	formate dehydrogenase-N subunit alpha	*Paracoccus haeundaensis*	100	WP_139598052.1			
CP157_03457	L-seryl-tRNA(Sec) selenium transferase	hybrid cluster	L-seryl-tRNA(Sec) selenium transferase	*Paracoccus* sp. 228	98	WP_045999769.1			
CP157_03458	Selenocysteine-specific elongation factor	hybrid cluster	selenocysteine-specific translation elongation factor	*Paracoccus* sp. S4493	98	WP_045983442.1			
CP157_03459	Leucine-responsive regulatory protein	hybrid cluster	Lrp/AsnC family transcriptional regulator	*Paracoccus* sp. PAMC22219	99	WP_042251546.1			

	Genome	AntiSMASH	pBLAST				Direct pBLAST		
**Locus Tag**	**Annotation**	**Cluster type**	**Description**	**Best hit**	**Similarity [%]**	**Accession**	**Reference gene**	**Similarity [%]**	**Coverage [%]**
CP157_03460	Bifunctional protein PutA	hybrid cluster	bifunctional proline dehydrogenase/L-glutamate gamma-semialdehyde dehydrogenase PutA	*Paracoccus marcusii*	99	WP_139086312.1			
CP157_03468	Alpha-D-kanosaminyltransferase, kanE	hybrid cluster	glycosyltransferase	*Paracoccus haeundaensis*	98	WP_139598126.1			
CP157_03469	Linear gramicidin synthase subunit D	hybrid cluster	amino acid adenylation domain-containing protein	*Paracoccus haeundaensis*	98	WP_139598060.1			
CP157_03474	3-oxoacyl-[acyl-carrier-protein] reductase FabG	hybrid cluster	oxidoreductase						
CP157_03475	6'''-hydroxyparomomycin C oxidase	hybrid cluster	GMC family oxidoreductase	*Paracoccus* sp. S4493	99	WP_045982069.1			
CP157_03477	Phthiocerol/phenolphthiocerol synthesis polyketide synthase type I PpsE	hybrid cluster	SDR family NAD(P)-dependent oxidoreductase	*Paracoccus haeundaensis*	99	WP_139598128.1			
CP157_03478	N-acetylglucosaminyl-diphospho-decaprenol L-rhamnosyltransferase	hybrid cluster	glycosyl transferase	*Paracoccus haeundaensis*	99	WP_139598065.1			
CP157_03479	Methionyl-tRNA formyltransferase	hybrid cluster	LLM class flavin-dependent oxidoreductase	*Paracoccus* sp. Arc7-R13	98	WP_127899508.1			
CP157_03480	dimodular nonribosomal peptide synthase	hybrid cluster	LLM class flavin-dependent oxidoreductase	*Pseudomonas stutzeri*	99	WP_148911014.1			
CP157_03481	Putative mycofactocin biosynthesis	hybrid cluster	glycosyltransferase family 2 protein	*Paracoccus* sp. S4493	99	WP_045983633.1			

This is a provisional file, not the final typeset article

Genome		AntiSMASH	pBLAST				Direct pBLAST		
Locus Tag	Annotation	Cluster type	Description	Best hit	Similarity [%]	Accession	Reference gene	Similarity [%]	Coverage [%]
	glycosyltransferase MftF								
CP157_03482	hypothetical	hybrid cluster	Cps/CapB family ryrosine-protein kinase	*Paracoccus haeundaensis*	99.6	WP_045983632.1			
CP157_03483	Chromosome partition protein Smc	hybrid cluster	lipopolysaccharide biosynthesis protein	*Paracoccus sulfuroxidans*	37	WP_145397099.1			
CP157_03484	Bifunctional hemolysin/adenylate cyclase	hybrid cluster	hemolysin type calcium-binding protein	*Pseudomonas stutzeri*	99	TYP67836.1			
CP157_03485	N-acetyl-alpha-D-glucosaminyl L-malate synthase	hybrid cluster	glycosyltransferase	*Paracoccus marcusii*	99	MQN30027.1			
CP157_03486	Alpha-maltose-1-phosphate synthase	hybrid cluster	glycosyltransferase involved in cell wall biosynthesis	*Pseudomonas stutzeri*	99	TYP67838.1			
CP157_03487	Undecaprenyl phosphate N,N'-diacetylbacillosamin e 1-phosphate transferase	hybrid cluster	sugar transferase	*Paracoccus* sp. 228	100	KIX18532.1			
CP157_03489	4'-phosphopantetheinyl transferase Npt	hybrid cluster	4'-phosphopantetheinyl transferase superfamily protein	*Paracoccus* sp. 228	97	WP_157020423.1			
CP157_03490	putative sensor histidine kinase pdtaS	hybrid cluster	two-component sensor histidine kinase	*Pseudomonas stutzeri*	99	TYP67842.1			
CP157_03498	hypothetical	bacteriocin	glycosyltransferase	*Paracoccus* sp. 228	98	WP_045999744.1			
CP157_03499	UDP-N-acetylglucosamine--N-acetylmuramyl-(pentapeptide) pyrophosphoryl-undecaprenol N-	bacteriocin	UDP-N-acetylglucosamine--N-acetylmuramyl-(pentapeptide) pyrophosphoryl-undecaprenol N-	*Pseudomonas stutzeri*	97	WP_148911026.1			

Genome		AntiSMASH	pBLAST				Direct pBLAST		
Locus Tag	Annotation	Cluster type	Description	Best hit	Similarity [%]	Accession	Reference gene	Similarity [%]	Coverage [%]
	acetylglucosamine transferase		acetylglucosamine transferase						
CP157_03500	sucrose synthase	bacteriocin	HAD-IIB family hydrolase	*Paracoccus haeundaensis*	99	WP_139598078.1			
CP157_03501	hypothetical	bacteriocin	DUF2282 domain-containing protein	*Paracoccus* sp. Arc7-R13	99	WP_127899525.1			
CP157_03502	hypothetical (marked as core biosynthesis gene)	bacteriocin	DUF692 domain-containing protein	*Paracoccus* sp. 228	99	WP_045999740.1			
CP157_03503	hypothetical	bacteriocin							
CP157_03504	hypothetical	bacteriocin	DoxX family membrane protein	*Paracoccus haeundaensis*	98	WP_139598082.1			
CP157_03505	ECF RNA polymerase sigma factor SigF	bacteriocin							
CP157_03506	Anti-sigma-F factor NrsF	bacteriocin	DUF1109 family protein	*Paracoccus haeundaensis*	99	WP_139598084.1			
CP157_03507	Putative L-lactate dehydrogenase operon regulatory protein	bacteriocin							

This is a provisional file, not the final typeset article

Supplement T 1: List of hemolysin genes found in the genome of CP157, their locus tags, results from pBLAST against the NCBI database and matches with UniProt entries for most similar known hemolysin genes.

Genome		pBLAST				UniProt		
**Locus Tag**	**Annotation**	**Description**	**Best Hit**	**Similarity [%]**	**Accesion**	**Entry**	**Reference organism**	**pBLAST (% similarity)**
CP157_00327	Bifunctional hemolysin/adenylate cyclase	Ca2+-binding RTX toxin-like protein	*Pseudomonas stutzeri*	94	TYP68132.1	J7QLC0	*Bordetella pertussis*	30
CP157_01338	Hemolysin, chromosomal	calcium-binding protein	*Paracoccus* sp. 228	100	KIX19514.1	P09983	*Escherichia coli*	48
CP157_01536	Hemolysin C	HlyC/CorC family transporter	*Paracoccus* sp. S4493	100	WP_045984219.1	Q68W10	*Rickettsia typhi*	33
CP157_02193	hypothetical	MULTIPSPECIES: thermostable hemolysin	*Proteobacteria*	98	WP_052714966.1			
CP157_02213	Leukotoxin	Hemolysin-type calcium-binding repeat-containing protein	*Paracoccus solventivorans*	57	SHL84061.1	P16462	*Aggregatibacter actinomycetemcomitans*	36
CP157_02217	Bifunctional hemolysin/adenylate cyclase	calcium-binding protein	*Paracoccus haeundaensis*	98	WP_139599722.1	J7QLC0	*Bordetella pertussis*	36
CP157_02231	Bifunctional hemolysin/adenylate cyclase	heme peroxidase	*Paracoccus haeundaensis*	99	WP_176695214.1	J7QLC0	*Bordetella pertussis*	27
CP157_02726	hemolysin-type calcium-binding region; frameshift	calcium-binding protein	*Paracoccus* sp. 228	98	WP_045999946.1			
CP157_02779	Bifunctional hemolysin/adenylate cyclase	family 16 glycosylhydrolase	*Paracoccus* sp. NBH48	97	WP_194886180.1	J7QLC0	*Bordetella pertussis*	63
CP157_03435	Bifunctional hemolysin/adenylate cyclase	calcium-binding protein	*Paracoccus haeundaensis*	99	TNH40245.1	J7QLC0	*Bordetella pertussis*	88
CP157_03484	Bifunctional hemolysin/adenylate cyclase	calcium-binding protein	*Paracoccus* sp. 228	99	WP_084693714.1	J7QLC0	*Bordetella pertussis*	98
CP157_03566	Bifunctional hemolysin/adenylate cyclase	calcium-binding protein	*Paracoccus haeundaensis*	47	WP_139599071.1	J7QLC0	*Bordetella pertussis*	94

Genome		pBLAST				UniProt		
Locus Tag	Annotation	Description	Best Hit	Similarity [%]	Accesion	Entry	Reference organism	pBLAST (% similarity)
CP157_03906	Bifunctional hemolysin/adenylate cyclase	calcium-binding protein	*Paracoccus* sp. SY	70	WP_146038606.1	J7QLC0	*Bordetella pertussis*	97

This is a provisional file, not the final typeset article
